# Detection and identification of Criegee intermediates from the ozonolysis of biogenic and anthropogenic VOCs: comparison between experimental measurements and theoretical calculations†
†Electronic supplementary information (ESI) available. See DOI: 10.1039/c7fd00025a


**DOI:** 10.1039/c7fd00025a

**Published:** 2017-03-06

**Authors:** Chiara Giorio, Steven J. Campbell, Maurizio Bruschi, Alexander T. Archibald, Markus Kalberer

**Affiliations:** a Department of Chemistry , University of Cambridge , Lensfield Road , Cambridge , CB2 1EW , UK . Email: chiara.giorio@atm.ch.cam.ac.uk ; Email: markus.kalberer@atm.ch.cam.ac.uk; b Dipartimento di Scienze dell’Ambiente e del Territorio e di Scienze della Terra , Università degli Studi di Milano Bicocca , Piazza della Scienza 1 , Milano , 20126 , Italy; c National Centre for Atmospheric Science , UK

## Abstract

Ozonolysis of alkenes is a key reaction in the atmosphere, playing an important role in determining the oxidising capacity of the atmosphere and acting as a source of compounds that can contribute to local photochemical “smog”. The reaction products of the initial step of alkene-ozonolysis are Criegee intermediates (CIs), which have for many decades eluded direct experimental detection because of their very short lifetime. We use an innovative experimental technique, stabilisation of CIs with spin traps and analysis with proton transfer reaction mass spectrometry, to measure the gas phase concentration of a series of CIs formed from the ozonolysis of a range of both biogenic and anthropogenic alkenes in flow tube experiments. Density functional theory (DFT) calculations were used to assess the stability of the CI-spin trap adducts and show that the reaction of the investigated CIs with the spin trap occurs very rapidly except for the large β-pinene CI. Our measurement method was used successfully to measure all the expected CIs, emphasising that this new technique is applicable to a wide range of CIs with different molecular structures that were previously unidentified experimentally. In addition, for the first time it was possible to study CIs simultaneously in an even more complex reaction system consisting of more than one olefinic precursor. Comparison between our new experimental measurements, calculations of stability of the CI-spin trap adducts and results from numerical modelling, using the master chemical mechanism (MCM), shows that our new method can be used for the quantification of CIs produced *in situ* in laboratory experiments.

## 1. Introduction

The Anthropocene has seen huge changes in the composition of the atmosphere. Gaseous, volatile organic compounds (VOCs) play an important role in determining the overall composition and reactivity of the atmosphere. Many VOC sources have significantly changed since the onset of the Anthropocene in strength but new sources and VOCs have also emerged. Oxidative degradation in the atmosphere with oxidants such as ozone is one of the main removal processes for VOCs. Olefinic VOCs react strongly with ozone leading to a complex reaction scheme with a large number of stable but also reactive intermediate reaction products. The initial step of alkene–ozone reactions is a 1,3-cycloaddition to produce a molozonide, which subsequently decomposes to produce so-called Criegee Intermediates (CIs) and a carbonyl product. In the condensed phase a further rearrangement is possible, but this is not the case in the gas phase.^1^–^3^RR′C=CR′′R′′′ + O_3_ → RR′COOOCR′′R′′′ → RRCOO + R′′C(O)R′′′

The formation of CIs was postulated over 40 years ago^4^ to proceed through the reaction of an alkene functional group with ozone (O_3_) but only in the last decade has our understanding of the short-lived CIs begun to flourish. The CI that is produced is thermally “hot” (sometimes referred to as an excited CI) and may undergo spontaneous decomposition to form other products^5^ or collisions with other molecules to lead to the stabilised CI (SCI).

The majority of recent studies that directly detected and studied SCI in the gas phase used a different route to its formation than the ozonolysis mechanism described above (*e.g.* Welz *et al.*,^6^ and references therein). Rather than reaction between O_3_ and unsaturated compounds, *gem*-iodo compounds have been shown to form CIs when photolysed in the presence of air:CH_2_I_2_ + *hν* (O_2_) → CH_2_OO + I + I

The formation of SCIs *via* this reaction has enabled new studies to probe the kinetics of bimolecular reactions that SCIs can undergo in the atmosphere. These studies have made use of a range of advanced laboratory techniques including photoionisation mass spectrometry and tunable synchrotron photoionisation mass spectrometry.^6^,^7^ Those techniques have been applied to the direct measurement of formaldehyde oxide, the simplest CI, and later on have made possible the discovery of the conformer-dependent reactivity of *syn*- and *anti*-acetaldehyde oxides,^8^ as these techniques are capable of distinguishing the two conformers from the difference in photoionisation energy. Subsequent studies detected formaldehyde oxide using near-UV cavity ring down spectroscopy,^9^ UV-Vis spectroscopy^10^–^12^ and IR spectroscopy.^13^ The latter was used also for the direct detection of the large β-pinene Criegee intermediate from an ozonolysis reaction.^14^

On the other hand, indirect measurements, exploiting the oxidation of SO_2_ to H_2_SO_4_ in the presence of an ˙OH scavenger, were used in Hyytiälä (a boreal forest in Finland) to quantify an oxidant “X” tentatively associated with SCIs with concentrations in the order of 5 × 10^4^ molecules per cm^3^.^15^,^16^ Other indirect methods exploited more specific reactions of organic reagents with CIs to identify their structure; Horie *et al.*^17^ found that hexafluoroacetone reacts rapidly with CIs to form compounds which are assignable to ozonides, 3,3-di(trifluoro)methyl-1,2,4-trioxolanes, which can be detected in FTIR spectroscopy. Very recently we presented a new cost-effective method to stabilise and detect CIs online in the gas phase by reacting them with spin traps and analysing the adducts that form using proton transfer reaction time of flight mass spectrometry (PTR-ToF-MS).^18^ This method was successfully applied to the measurement of CIs from the ozonolysis of α-pinene, the structure of the CI-spin trap adduct was characterised in detail and we showed the potential of this technique to be used for quantification purposes.^18^

Here we expand on our previous study^18^ by measuring CIs from the ozonolysis of a series of biogenic and anthropogenic VOCs such as β-pinene, limonene, methacrolein, *cis*-2-hexene, styrene and also a mixture of more than one olefinic precursor. Experimentally measured concentrations of CI-spin trap adducts are compared with those which are theoretically expected, and the differences are explained in terms of the stability of CI-spin trap adducts and instrumental response. We demonstrate that our new technique is uniquely capable of quantifying many different CIs simultaneously and thus provides a significant step towards studying CIs in realistic, complex reaction mixtures.

## 2. Materials and methods

### 2.1. Chemicals

For the gas phase ozonolysis experiments, the following VOC precursors were used: styrene (≥99.9%, Reagentplus®, Sigma Aldrich), *R*-(+)-limonene (≥99.0%, Sigma-Aldrich), methacrolein (≥95%, Aldrich), (–)-β-pinene (≥99.0%, Aldrich) and *cis*-2-hexene (≥95%, Aldrich). The spin trap 5,5-dimethyl-pyrroline N-oxide (DMPO) (≥97%, GC grade, Sigma Aldrich) was used to capture and stabilise CIs in the gas phase.

### 2.2. Flow tube set-up

**Fig. 1:**
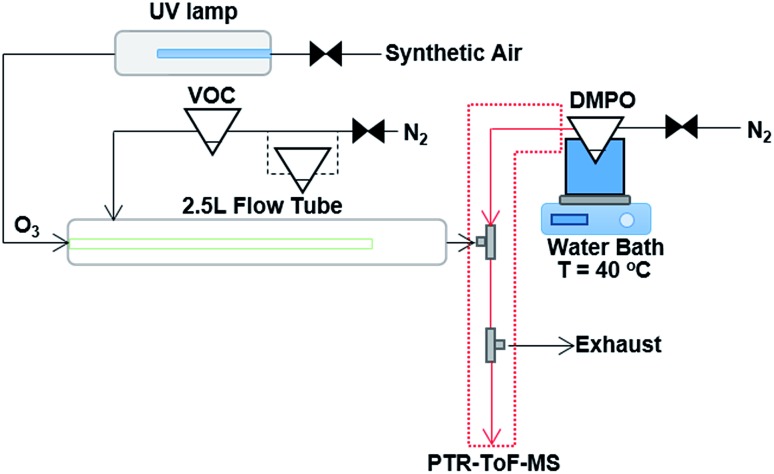
Schematic of the experimental set up, consisting of a 2.5 L glass flow tube where an olefinic precursor reacts with ozone, a mixing point (T-fitting) in which the spin trap is mixed with the sample flow from the flow tube, and a heated PTFE tube in which the spin trap reacts with the CI before detection and quantification with PTR-ToF-MS. For the experiments where two VOC precursors were mixed, an additional pear shaped flask was added in-line with the N_2_ carrier gas flow. Modified from Giorio *et al.*^18^

The experimental technique of using the spin trap DMPO to capture gas phase CIs with subsequent analysis of the adduct formed with PTR-ToF-MS has been described previously in detail.^18^ The ozonolysis reaction takes place in a flow tube reaction vessel, which is maintained at ambient temperature (∼16 to 18 °C) and pressure, and dry conditions (relative humidity < 2%) as shown in Fig. 1. The experimental set-up comprises of a 2.5 L glass flow tube, in which the olefinic precursor reacts with ozone with a reaction time of three seconds (see Fig. S1 in the ESI†) before the sample flow is mixed with a N_2_ flow containing the gaseous spin trap which scavenges and forms stable adducts with the CIs. A heated PTFE tube in which the spin trap reacts with the CI connects the mixing point with the PTR-ToF-MS for quantification.

The olefinic precursors were evaporated from a 25 mL pear-shaped flask filled with 0.5 mL of pure compound and introduced continuously in the flow tube using N_2_ (at 175 cm^3^ min^–1^, oxygen-free nitrogen, BOC) carrier gas regulated *via* a 20–2000 cm^3^ min^–1^ mass flow controller (MKS 1179A Mass-Flo® controller). For experiments with *cis*-2-hexene and methacrolein, due to the fact that they are more volatile compared to other VOC precursors, the pear shaped flask was submersed in a dry ice/acetonitrile bath (–41 °C) in order to maintain a lower steady-state concentration of these compounds in the flow tube. The other VOCs were maintained at ambient temperature. Ozone was produced by flowing synthetic air (Zero grade, BOC) past a UV lamp (185/254 nm, Appleton Woods®) at 155 cm^3^ min^–1^ (20–2000 cm^3^ min^–1^ MKS 1179A Mass-Flo® controller). The UV lamp used in this study produced a lower amount of ozone compared with the previous study on α-pinene ozonolysis,^18^ reaching a maximum concentration in our system of 18 ppm measured using a UV photometric ozone analyser (Thermo Scientific model 49i) and shown in Fig. S2.† The outlet of the flow tube is mixed into a T-connection (stainless steel 1/4′′ (∼6.35 mm) T-fitting, Swagelok®) with a 310 cm^3^ min^–1^ flow (50–5000 cm^3^ min^–1^ MKS 1179A Mass-Flo® controller) of DMPO in N_2_ (oxygen-free nitrogen, BOC) evaporated from a 25 mL flask filled with 0.5 mL of DMPO, which is held in a water bath at 40 °C. Connecting tubes and the T-connection were kept at 85 °C to avoid condensation of DMPO.

### 2.3. PTR-ToF-MS measurements

Online gas phase concentrations of the VOC precursors, DMPO and CI–DMPO adducts were measured using a proton transfer reaction time-of-flight mass spectrometer (PTR-ToF-MS 8000, Ionicon Analytik, Innsbruck, Austria) in the *m*/*z* range 10–500, with a time resolution of 10 s and a mass resolution *m*/Δ*m* of approximately 5000 (full width at half maximum) at the mass of protonated acetone. Source settings for all experiments were: a drift tube pressure ∼2.22 mbar, a drift tube voltage of 510 V and a drift tube temperature = 90 °C; resulting in an *E*/*N* of ∼127 *T*_d_ (1 *T*_d_ = 10^–17^ V cm^2^). The PTR-ToF-MS inlet (a 1 m long inert PEEK tube, ID = 1 mm, OD = 1.59 mm) was kept at 100 °C and the sampling flow rate was 100 cm^3^ min^–1^. Data analysis was conducted using PTR-MS Viewer 3.2 (Ionicon Analytik). The concentration of the olefinic precursors were estimated on the basis of the rate constants of the proton transfer reaction, which were: 2.33 × 10^–9^ cm^3^ per molecule per s (styrene), 2.54 × 10^–9^ cm^3^ per molecule per s (limonene), 3.55 × 10^–9^ cm^3^ per molecule per s (methacrolein) and 2.50 × 10^–9^ cm^3^ per molecule per s (β-pinene).^19^ The value for *cis*-2-hexene is unknown, so it was approximated to be the value for the isomer *trans*-2-hexene (2.05 × 10^–9^ cm^3^ per molecule per s).^19^ For DMPO and the CI–DMPO adducts, ion–polar molecule capture collision rate constants were calculated as detailed in Section S4 and elsewhere.^20^,^21^

For quantification of the initial concentrations of β-pinene and limonene both the protonated molecular ion C_10_H_17_^+^ and the fragment C_6_H_9_^+^ were used, for *cis*-2-hexene both the protonated molecular ion C_6_H_13_^+^ and the main fragment C_3_H_7_^+^, for methacrolein the protonated molecular ion C_4_H_7_O^+^ and the fragments C_3_H_7_^+^ and C_3_H_5_^+^ while for styrene, only the protonated molecular ion C_8_H_9_^+^ was used for quantification.

DMPO and VOC signals are often in saturation during the experiments and therefore the corresponding ^13^C isotopes were used for quantification. The initial concentrations of VOCs and DMPO were also evaluated by diluting the sample flow with pure N_2_ in a ratio of 1 : 10 as detailed in Giorio *et al.*^18^

### 2.4. DFT calculations

Geometry optimizations and energy calculations have been carried out in the density functional theory (DFT) framework with the TURBOMOLE 6.4 suite of programs^22^ by using the BP86 (ref. 23 and 24) and B3LYP^25^–^27^ functionals, and a valence triple-ζ basis set with polarization functions on all atoms (TZVP).^28^ For the BP86 functional the resolution-of-the-identity (RI) technique is applied.^29^ As the geometries and the energy differences calculated by the two functionals are qualitatively similar and give the same interpretation of the results in the Section 3.2 only B3LYP calculations will be discussed (see Fig. 3 and Table S1† for a comparison of the BP86 and B3LYP results). Stationary points of the energy hypersurface have been located by means of energy gradient techniques and full vibrational analysis has been carried out to further characterise each stationary point and for the calculation of the thermochemical corrections for determining enthalpies at 298 K. The optimization of transition state structures has been carried out according to a procedure based on a pseudo Newton–Raphson method. The search for the transition state structure is carried out using an eigenvector-following algorithm in which, the search is performed by choosing the critical eigenvector with a maximum overlap criterion, which is based on the dot product with the eigenvector followed at the previous step. Finally, the analytical Hessian matrix is calculated to carry out vibrational analysis of the stationary point. Energies of the van der Waals complexes have been corrected for the basis set superposition error using the procedure of Boys and Bernardi.^30^ Average static polarisabilities have been calculated at the B3LYP/def-TZVP level of theory by using the first-order response theory as implemented in TURBOMOLE 6.4.^31^,^32^

### 2.5. MCM modelling

We compare our experimental results with modelled time evolutions of SCIs using the AtChem (https://atchem.leeds.ac.uk/) numerical box-model. AtChem is developed for use with the Master Chemical Mechanism (MCM).^33^ Model input parameters used in all simulations are included in Table 1. In total, we performed six simulations using initial conditions from Table 1 and the mixing ratios of O_3_ and VOCs as measured in our flow tube experiments. AtChem was run on-line, making use of the most recent version (v1.5). The numerical model makes use of the Fortran CVODE library for the integration of the stiff ODEs that are represented by the MCM reaction mechanism. For each AtChem simulation we downloaded a unique MCM input file. This input file contained all the relevant inorganic and organic chemical reactions that were integrated forward in time by AtChem.

**Table 1:** Parameters and their values used in the AtChem box-model simulations of our flow tube experiments

Parameter	Value (units)
Temperature	289.15 K
Number density (M)	2.60 × 10^19^ (molecules per cm^3^)
[H_2_O]	1.23 × 10^14^ (molecules per cm^3^)
[O_3_]	4.41 × 10^14^ (molecules per cm^3^)

## 3. Results and discussion

**Fig. 2:**
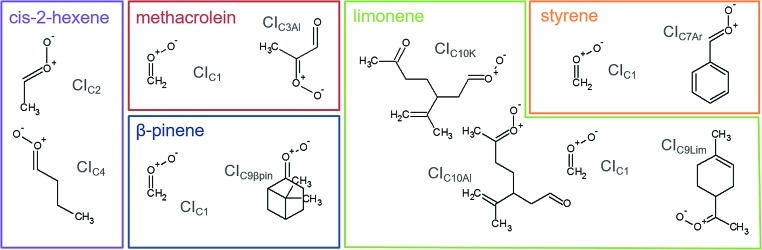
Molecular structures and acronyms of the CIs detected from the ozonolysis of *cis*-2-hexene, methacrolein, β-pinene, styrene and limonene.

We used stabilisation with the spin trap DMPO and analysis with PTR-ToF-MS to quantify the CIs-adducts produced from the ozonolysis of five VOCs (Fig. 2). The VOCs under study have been chosen as representative of different classes of compounds of interest in atmospheric chemistry, including biogenic VOCs, such as β-pinene, limonene, methacrolein, and anthropogenic VOCs, such as *cis*-2-hexene and styrene. Among these, methacrolein also represents oxidised VOCs and styrene represents aromatic olefins. The objective of this study is to assess the quantification capability of our new measurement technique. To do so, we used theoretical calculations to investigate the mechanism of formation of the CI–DMPO adducts, the energy barriers of these reactions and assess the stability of the adducts. Subsequently, we performed ozonolysis experiments of the VOCs in a flow tube to detect and quantify the CI–DMPO adducts from the five individual VOCs and from a mixture of two different VOCs (namely β-pinene and *cis*-2-hexene). Additionally, we compared the experimentally measured concentrations of CI–DMPO adducts with those expected from numerical modelling, using the AtChem/MCM model.

### 3.1. Mechanism of formation and stability of CI–DMPO adducts

The measurement method used in this study, presented for the first time in Giorio *et al.*,^18^ is based on the stabilisation of the highly reactive CIs using the spin trap DMPO. Assessing the quantification capability can be challenging as the kinetics of formation of the CI–DMPO adducts and the stability of such adducts are unknown. To support and aid interpretation of our experimental results, the stability of the CI–DMPO adducts generated by the ozonolysis of β-pinene, *cis*-2-hexene and methacrolein, and the mechanism and energy barriers of their formation have been investigated by DFT calculations (see Fig. 2 for the CIs).

**Fig. 3:**
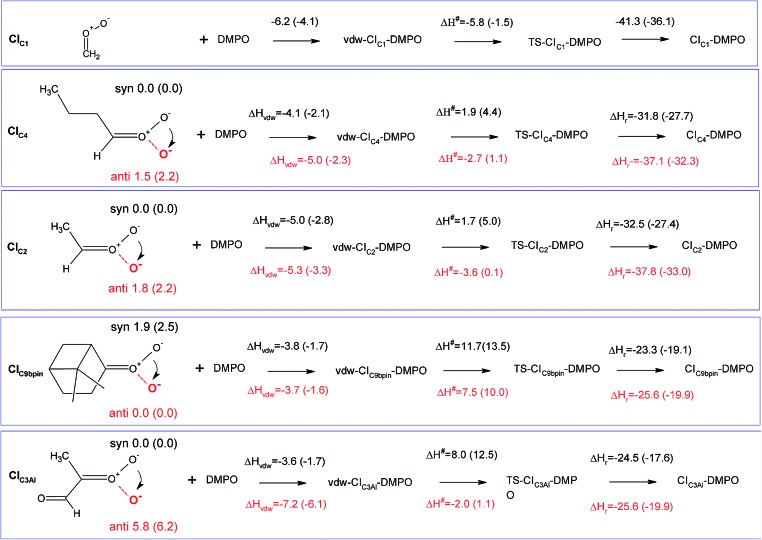
Schematic representation of the reaction of CIs with DMPO with values of reaction and activation enthalpies. The first step for each reaction is the formation of the van der Waals adduct (vdw-CI–DMPO), the second step is the formation of the transition state (TS-CI–DMPO) and the last step is the conversion to the final adduct (CI–DMPO). All values refer to enthalpy differences calculated with respect to the separated reactants using the B3LYP functional. In parenthesis are the corresponding values calculated using the BP86 functional. The molecular structures define the *anti* (red terminal oxygen) and the *syn* (black terminal oxygen) conformers. The values over (in black) and under (in red) the arrows refer to the reaction of the *syn* and *anti* conformers, respectively.

An extensive search on the potential energy surface (PES) of these CIs was carried out to identify the relevant minimum energy conformations. It turns out that for CI_C2_, CI_C4_ and CI_C3Al_ the *syn* conformation is more stable than the *anti* one by 1.8, 1.5 and 5.8 kcal mol^–1^, respectively, whereas in CI_C9βpin_ the *anti* conformer is more stable than the *syn* conformer by about 2 kcal mol^–1^ (*n.b.* hereafter we will refer to the *syn* conformation as that in which the outer oxygen points toward the alkyl group in CI_C2_, CI_C4_ and CI_C3Al_, and toward the less H-rich C(CH_3_)_2_ group in CI_C9βpin_; see Fig. 3 for the definition of *syn* and *anti* conformations). It is worth noting that these results are in good agreement with previous calculations carried out using *ab initio* highly correlated methods.^34^–^37^ The addition of CIs with DMPO have been investigated by taking into account both *anti* and *syn* conformers to inspect potential differences in the reactivity of the two species.

The cycloaddition of the CIs to the spin-trap DMPO can occur through the attack of the carbon atom of the CI on either the nitrogen or the oxygen atoms of the DMPO nitrone group leading to the formation of a 5-membered or a 6-membered ring, respectively. DFT calculations show that for all of the compounds investigated, the 6-membered ring species is much more stable than the corresponding 5-membered ring species (see Table S1†), in agreement with the results obtained from the investigation of the formation of CI–DMPO adducts from the ozonolysis of α-pinene.^18^ Therefore, in the following, only the addition of CIs to DMPO to give 6-membered ring adducts will be considered.

The first step in the reaction of the CIs with DMPO is the formation of pre-reactive van der Waals adducts in which the carbonyl oxide approaches the nitrone group of DMPO (see Fig. S9–S13†). The interaction energies of such van der Waals complexes are within –3.6 and –7.2 kcal mol^–1^, with the two extremes given by CI_C3Al_ in the *syn* and *anti* conformations (see Fig. 3).

The reactivity of the CIs strongly depends on the number of the substituents attached to the carbon atom of the carbonyl oxide, and on the initial conformation of the CI reactants (see Fig. 5 and 6). The reaction of the parent formaldehyde oxide CI_C1_ with DMPO is barrierless as the activation enthalpy is lower than the enthalpy of the separated reactants (Δ*H*^#^ = –5.8 kcal mol^–1^), and only 0.5 kcal mol^–1^ higher than that of the van der Waals adduct. This reaction is also strongly exothermic with the value of the reaction enthalpy (Δ*H*_r_) as low as –41 kcal mol^–1^. The energy barriers for the addition to DMPO of CI_C2_ and CI_C4_, featuring one alkyl substituent bounded to the carbon atom of the carbonyl oxide, are slightly larger than that calculated for the parent CI_C1_, and the reactions are slightly less exothermic. Indeed, when considering the most stable *syn* conformers (CI_C2(*syn*)_ and CI_C4(*syn*)_) as reactants, the energy barriers for both species are about 2 kcal mol^–1^ with respect to the separated reactants, and about 7 kcal mol^–1^ with respect to the van der Waals complexes. The reaction enthalpies of these two cycloadditions (Δ*H*_r_) are also very similar and equal to about –32 kcal mol^–1^. The reaction is still more favoured when starting from the less stable *anti* conformers (CI_C2(*anti*)_ and CI_C4(*anti*)_). In this case the Δ*H*^#^ is negative by 3 kcal mol^–1^ with respect to the separated reactants, and only 2 kcal mol^–1^ higher than the van der Waals complexes. The Δ*H*_r_ is lower than that of the *syn* conformer (Δ*H*_r_ = –38 kcal mol^–1^) due to the fact that the reactants are higher in energy, and that the ring closure of CI_C2(*anti*)_ and CI_C4(*anti*)_ yields the RR/SS diastereoisomers which are more stable than the RS/SR ones formed by the ring closure of CI_C2(*syn*)_ and CI_C4(*syn*)_.

The activation enthalpy calculated for the cycloaddition of CI_C9βpin_ in the most stable *syn* conformation is equal to 11.7 kcal mol^–1^, a value significantly larger than that calculated for the CIs discussed above. Correspondingly, the Δ*H*_r_ is equal to about –24 kcal mol^–1^, more than 10 kcal mol^–1^ higher than that calculated for the other CIs. The reaction of the less stable *anti* conformer has an energy profile similar to that of the *syn* conformer with Δ*H*^#^ and Δ*H*_r_ equal to –8 and –26 kcal mol^–1^, respectively. The difference in reactivity of CI_C9βpin_ compared to the other CIs may be due to the connectivity of the carbon atom of the carbonyl oxide, which in CI_C9βpin_ is bound to two other carbons. It is worth noting that the same trend in activation and reaction energies was observed for the addition of DMPO to the two CIs generated by the ozonolysis of α-pinene that we have previously investigated.^18^ The two adducts have one and two alkyl substituents attached to the carbon atom of the carbonyl oxide, and feature energy barriers and reaction energies that differ by more than 10 kcal mol^–1^ in favour of the less substituted species.

CI_C3Al_ is the species featuring the largest difference in the reactivity of the *syn* and *anti* conformers. Considering the most stable *syn* conformer, it turns out that Δ*H*^#^ and Δ*H*_r_ are equal to 8.0 and –25 kcal mol^–1^, which are values similar to those calculated for CI_C9βpin_. On the other hand, considering the less stable *anti* conformer, the energy profile is much more favourable as the barrier is equal to about –2 kcal mol^–1^ with respect to the separated reactants (+5.2 kcal mol^–1^ with respect to the van der Waals complex) and the Δ*H*_r_ is equal to –33 kcal mol^–1^, values that fit better those calculated for CI_C2_ and CI_C4_.

**Fig. 4:**
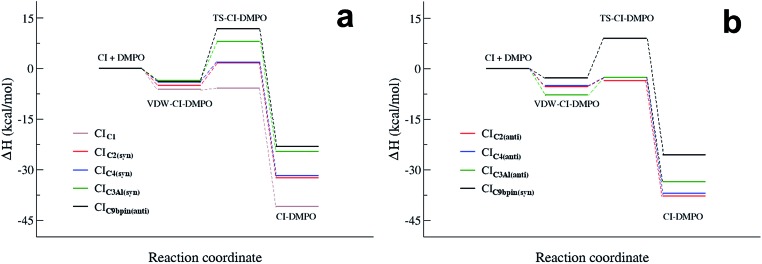
Reaction energy profiles of the CI + DMPO → CI–DMPO reactions of CI_C1_, CI_C2_, C_IC4_, C_9βpin_ and CI_C3Al_, calculated starting from the more stable (a) and the less stable (b) conformation of each adduct.

Fig. 4 summarizes the results presented above showing the reaction energy profiles for the formation of the CI–DMPO adducts starting from the CIs in the more stable (Fig. 4a) and less stable (Fig. 4b) conformations. These results show that the reaction of the investigated CIs with DMPO occurs very rapidly, with the exception of CI_C9βpin_ for which, in both conformations the activation energies are larger than those calculated for the other CIs. In addition, all the reactions are strongly exothermic, but with an Δ*H*_r_ value that becomes significantly less negative upon increasing the number of substituents of the carbonyl oxide carbon atom (*i.e.* the Δ*H*_r_ of the CI_C9βpin_–DMPO adduct is more than 15 kcal mol^–1^ higher than that of CI_C1_–DMPO).

It also worth noting that for all the CIs investigated the less stable conformer has a more favourable energy profile. In particular, CI_C3Al_, which is characterized by the larger difference in stability of the conformers, also features a larger change in the reactivity of such conformers. Similar behaviour has been observed experimentally by Taatjes *et al.*^8^ who reported that the *anti* (less stable) conformer of acetaldehyde oxides is more reactive than the *syn* one with both H_2_O and SO_2_.

### 3.2. Detection of CIs in the gas phase from biogenic and anthropogenic VOCs

**Fig. 5:**
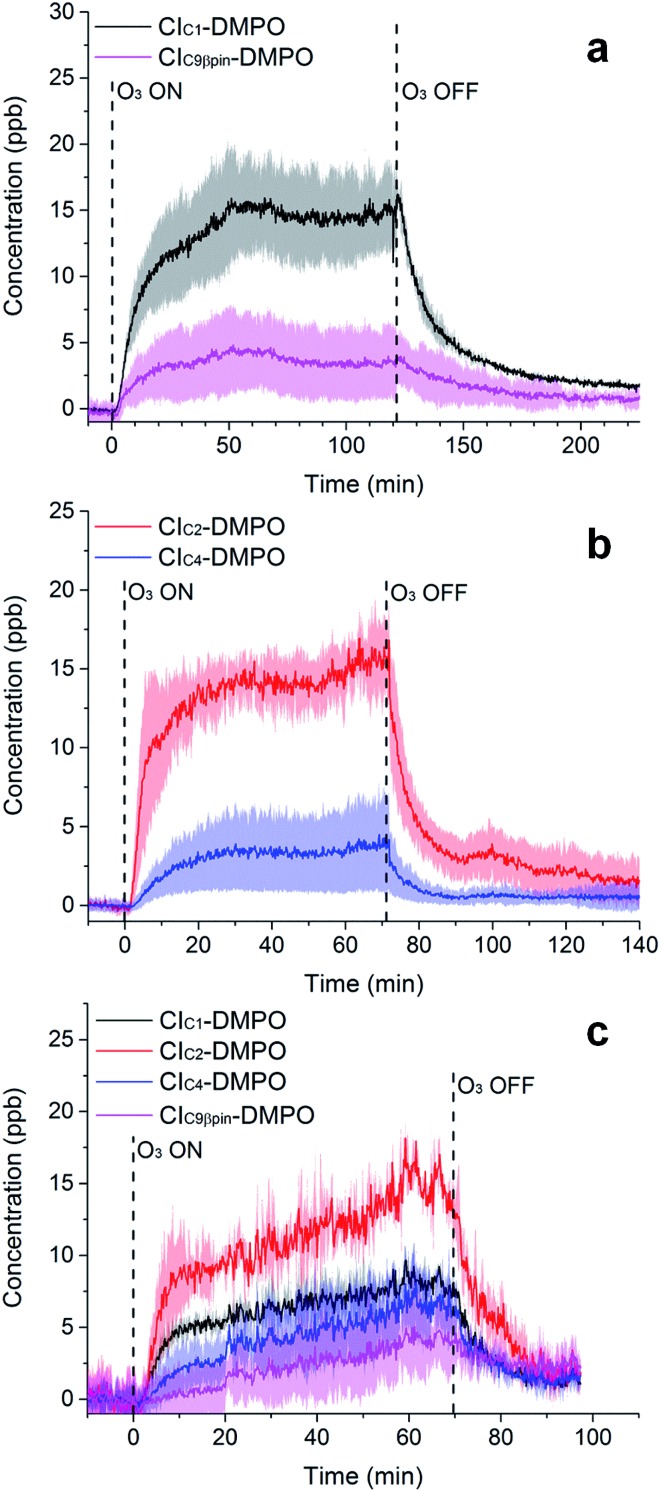
Time-series of CIs formed from the ozonolysis of β-pinene (a), *cis*-2-hexene (b) and a mixture of β-pinene and *cis*-2-hexene (c) in a steady state flow tube reaction system.

The adducts formed between the spin trap DMPO and the β-pinene CIs, with the elemental formulas C_15_H_26_NO_3_^+^ (*m*/*z* = 268.1907) and C_7_H_14_NO_3_^+^ (*m*/*z* = 160.0968), were detected by the PTR-ToF-MS (Fig. 5a) using the optimised conditions described above. This is consistent with previous studies using IR detection of CIs^14^ and our previous work on α-pinene^18^ in which CI–DMPO with the elemental formula C_16_H_28_NO_4_^+^, was detected at *m*/*z* 298.2013 (the two expected α-pinene CIs are indistinguishable in MS, as the double bond is in the *endo* position and the two CIs have the same mass). The reaction mechanism of the formation of the CI–DMPO adduct was elucidated in our previous study^18^ and theoretical calculations were used to assess the CI-specific stability of the spin trap adducts (Section 3.1).

The observed concentrations of the two CI–DMPO adducts are about three to four orders of magnitude lower compared with the initial concentration of the reagents, which were 18, 83 and 110 ppm for O_3_, β-pinene and DMPO, respectively. The concentrations of CI–DMPO adducts are also about three and four orders of magnitude lower compared with the steady-state concentration of β-pinene (Table 2) which is in excess with respect to ozone. Notably, ozone can react not only with alkenes but also with the spin trap DMPO, therefore decreasing its concentration and decreasing the efficiency of the spin trapping reaction. For this reason, in the series of experiments that we report here, ozone concentration was lower than in the previous experiments performed with α-pinene^18^ and in most of the cases was the limiting reagent, in order to minimise losses of DMPO (see VOC concentrations in Table 2).

**Table 2:** Initial and steady-state concentrations of VOCs in our experiments and comparison between experimental measurements and expected concentrations of CIs from MCM modelling for all the VOCs examined in this study and the VOC mixture

	Measured^*a*^ [VOC]_0_ (ppm)	Measured^*b*^ [VOC]ss (ppm)	CIs–DMPO	Measured^*c*^ [CIs–DMPO]ss (ppb)	Measured ratios [CIs–DMPO] : [VOC]_0_	Measured fraction (%) of CIs–DMPO^*c*^	Modelled [SCIs] (ppb)	Modelled fraction (%) of SCIs
**VOCs**								
β-Pinene	83	65 ± 2	CI_C1_–DMPO	14.8 ± 3.6	2 × 10^–4^	79.6 ± 33.5	250	59.1
			CI_C9βpin_–DMPO	3.8 ± 2.8	5 × 10^–5^	20.4 ± 16.6	173	40.9
*cis*-2-Hexene	120	24 ± 1	CI_C2_–DMPO	14.5 ± 1.9	1 × 10^–4^	80.6 ± 22.7	875	50
			CI_C4_–DMPO	3.5 ± 2.6	3 × 10^–5^	19.4 ± 15.2	875	50
Methacrolein	838	369 ± 419	CI_C1_–DMPO	8.7 ± 1.3	1 × 10^–5^	84.5 ± 19.4	334	93.8
			CI_C3Al_–DMPO	1.6 ± 0.5	2 × 10^–6^	15.5 ± 5.6	22	6.2
Limonene	6.3	1.4 ± 0.3	CI_C1_–DMPO	7.8 ± 0.6	1 × 10^–3^	42.2 ± 10.1	0.5	0.3
			CI_C9Lim_–DMPO	7.2 ± 2.8	1 × 10^–3^	38.9 ± 17.5	0	0
			CI_C10K/C10Al_–DMPO	3.5 ± 0.8	6 × 10^–4^	18.9 ± 6.1	169^*d*^	99.7
Styrene	78	8 ± 3	CI_C1_–DMPO	18.6 ± 5.2	2 × 10^–4^	93.5 ± 38.4	191	50
			CI_C7Ar_–DMPO	1.3 ± 0.8	2 × 10^–5^	6.5 ± 4.5	191	50

**mixVOCs**								
β-Pinene	96	55 ± 13	CI_C1_–DMPO	6.8 ± 1.1	7 × 10^–5^	24.3 ± 7.8	101	5.1
			CI_C9βpin_–DMPO	2.6 ± 2.4	3 × 10^–5^	9.3 ± 9.0	69	3.5
*cis*-2-Hexene	153	88 ± 6	CI_C2_–DMPO	11.8 ± 1.6	8 × 10^–5^	42.1 ± 13.1	897	45.7
			CI_C4_–DMPO	4.9 ± 2.2	3 × 10^–5^	17.5 ± 9.3	897	45.7

^*a*^Concentration measured in dilution experiments.

^*b*^Experimental uncertainty expressed as standard deviation between 2–3 repeated experiments. Larger uncertainties affect the most volatile VOCs for difficulties in maintaining a constant gas phase concentration in our experimental set-up.

^*c*^Experimental uncertainty expressed as standard deviation between 2–3 repeated experiments. It does not take into account systematic errors due to unknown fragmentation pattern.

^*d*^Referred to SCI_C10K_.

**Fig. 6:**
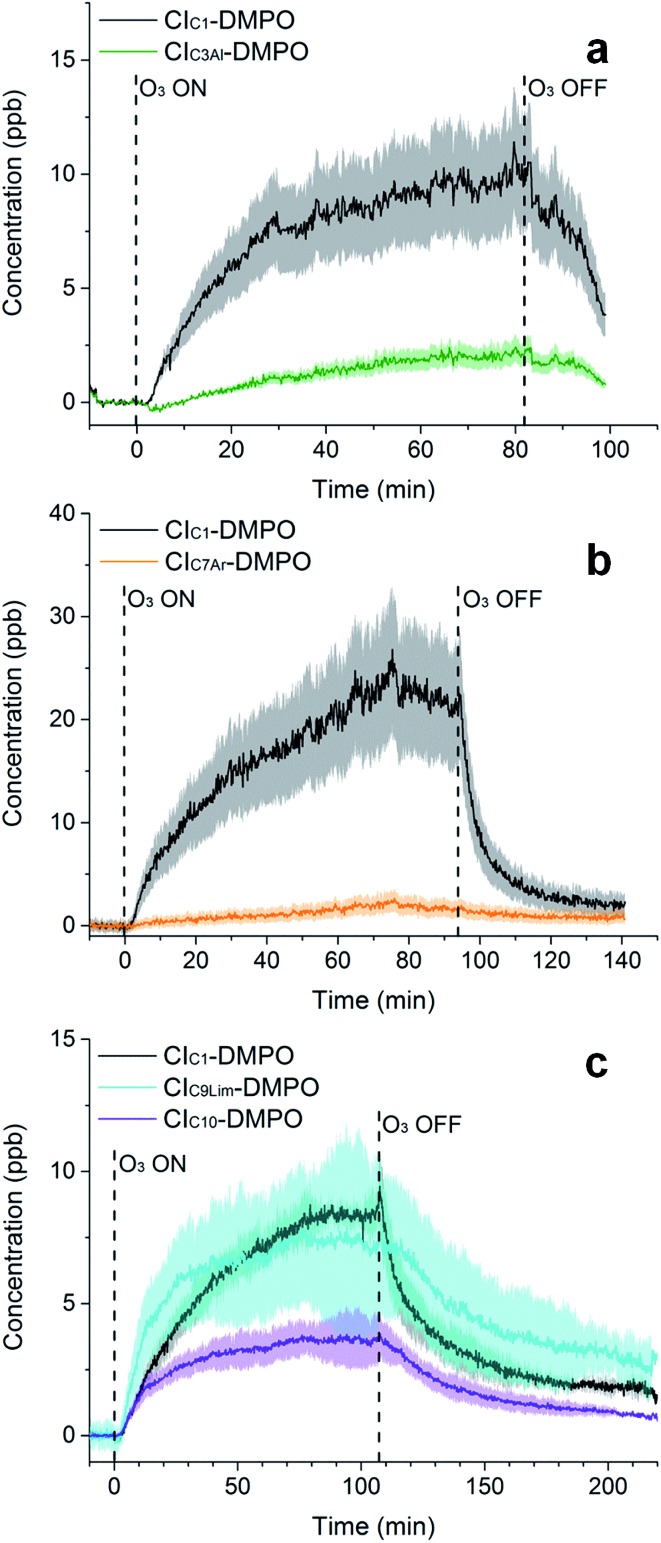
Time-series of CIs formed from the ozonolysis of methacrolein (a), styrene (b) and limonene (c) in a steady-state flow tube reaction system. For limonene ozonolysis, the CI_C10Al_ and CI_C10K_ have the same exact mass, therefore are indistinguishable in MS and are reported here as one time trace (CI_C10_).

The concentrations of the CI–DMPO adducts obtained are stable over time scales of one hour or more in the steady-state flow tube set up used here (Fig. 5 and 6) and are well reproducible in this system, with a variation of ±25% on average observed in multiple repeats. The slow initial increase in CI–DMPO concentration is likely associated with the varying amount of O_3_ produced from the UV lamp. In fact, the UV lamp has a warm up time of about 20–30 minutes in which ozone concentration exponentially increases before reaching a plateau (Fig. S2†). After the UV lamp is switched off, the concentration of CI–DMPO adducts decreases slowly to zero, probably due to memory effects as the DMPO and its adducts can condense on the walls of the tubing.

Similarly, for *cis*-2-hexene the two expected CI–DMPO adducts with molecular formulas C_10_H_20_NO_3_^+^ and C_8_H_16_NO_3_^+^ have been detected at *m*/*z* 202.1438 and 174.1125, respectively and likewise, they are stable over time in the steady-state reaction system (Fig. 5b). Additional experiments in which both β-pinene and *cis*-2-hexene have been concurrently injected in the flow tube have been carried out. Also in this case, all four expected CIs from both VOCs have been detected with good repeatability as shown in Fig. 5c. To the authors’ knowledge, this is the first time in which detection and identification of CIs from multiple VOC precursors has been achieved, clearly demonstrating the capability of this technique to characterise CIs in complex, atmospherically relevant VOC mixtures. Concentrations of CIs from *cis*-2-hexene are higher than the concentrations of CIs from β-pinene (Table 2) which is consistent with the higher initial concentration of *cis*-2-hexene (153 ppm) than that of β-pinene (96 ppm).

Furthermore, the study has been additionally extended to other VOCs with different chemical properties and volatilities. Methacrolein, a first-generation oxidation product from isoprene, has been ozonolysed in the flow tube and the two expected CIs have been detected, the CI_C1_–DMPO and the aldehydic CI_C3Al_–DMPO (Fig. 6a). Also for styrene, an aromatic olefin, both the CI_C1_–DMPO and the aromatic CI_C7Ar_–DMPO have been detected (Fig. 6b).

Concerning limonene, a diene monoterpene, all CIs from the reaction of ozone with both the *endo*- and *exo*-double bond have been detected (Fig. 6c). From the comparison between the rate of the reaction of ozone with limonene and that of ozone with limononaldehyde, and the low yields of limona ketone, the ozonolysis of limonene should occur predominantly at the *endo*-double bond (95 : 5).^38^ However, ozone was in excess in our conditions (18 ppm of ozone and 6 ppm of limonene) which can explain the high concentration of CIs detected from the less favoured reaction channel. No second generation CIs were detected from the reaction of ozone with an olefinic first-generation oxidation product, but these CIs are likely to be very low volatility compounds and they probably partition quickly into the condensed phase.

In general, the detected mixing ratios of CIs are between three and five orders of magnitude lower than the initial concentrations of the olefinic precursor and between two to four orders of magnitude lower than the measured concentration of olefinic precursor at the steady state (see Table 2). The concentration of olefinic precursors is generally in excess with respect to ozone, except for limonene. During the three seconds reaction time in the flow tube, CIs can decompose to form a wide range of further products, including dioxiranes and vinylhydroperoxides which retain the same molecular mass as the CIs. According to the reaction mechanism proposed by Adam *et al.*^39^ the reaction of dioxirane with DMPO yields a product with a mass different to the CI–DMPO adducts. As pointed out by Liu *et al.*^40^ the vinylhydroperoxide forms with a significant excess energy and rapidly undergoes O–O bond fission to form ˙OH. Nevertheless, the presence of organic acids may help to dissipate the excess energy and stabilise this species so it has to be assessed in future studies whether the vinylhydroperoxide may interfere to some degree with the measurement.

Because of the high VOC concentrations used here, their instrumental signals are likely outside of the linear range of the instrument and therefore the steady-state concentrations derived may be lower limits of their actual concentrations in the flow tube. Other factors should be optimised and characterised for an improved quantification of the CI-adducts, including the effect of secondary organic aerosol formation in the flow tube, wall losses throughout the system, the unknown kinetics of the CI-spin trap reactions, and unknown fragmentation patterns of the CI–DMPO adducts in the mass spectrometer.

To the authors’ knowledge, this is the first time in which detection of CIs from methacrolein, limonene, styrene and *cis*-2-hexene is achieved, and the first time in which four CIs from a mixture of two olefinic precursors were simultaneously detected.

### 3.3. Comparison between experimental measurements and MCM modelling

To compare our experimental results and test the quantification capability of the technique in our experimental conditions, experimental results have been compared with MCM model simulations. The complexity of the entire ozonolysis reaction scheme is vast, and a plethora of compounds produced in this system can scavenge CIs, including the carbonyl compound produced in a 1 : 1 ratio during the first steps of the reaction. Whilst not a fully explicit chemical mechanism, the MCM can help in understanding the complexity of the system and evaluate the amount of both excited and stabilised CIs available at the outlet of the flow tube (3 seconds reaction time) in the experiments performed.

**Fig. 7:**
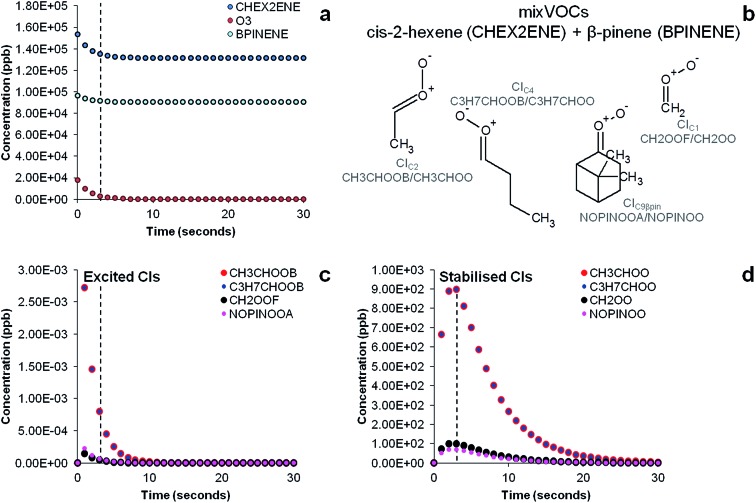
Time evolution of precursors (a), structures and acronyms of CIs (b) and time evolution of excited CIs (c) and stabilised CIs (d) in the ozonolysis of a VOC mixture of β-pinene and *cis*-2-hexene determined by the MCM model simulating our experimental conditions. Dashed vertical bars indicate reaction time (3 seconds) in our steady-state flow tube experiments during which ozone reacts with the olefinic precursors producing CIs before they are mixed with the DMPO.

The results of the AtChem/MCM modelling simulating the experiment of ozonolysis of the VOC mixture containing β-pinene and *cis*-2-hexene are reported in Fig. 7. The results show the decay of β-pinene, *cis*-2-hexene and ozone with a time resolution of one second (Fig. 7a) in which it can be seen that after a three seconds reaction time in the flow tube, concentrations of β-pinene and *cis*-2-hexene are still very high as ozone is not in excess and its concentration in turn rapidly decreases. It can also be seen in Fig. 7c that excited CIs decompose quickly in the flow tube and their concentrations in our steady-state reaction system are lower than the detection limits (∼30 ppt).^18^ Conversely, detectable amounts in the ppb range of SCIs are still present at the end of the flow tube (Fig. 7d) and can therefore be detected by our technique.

The results of the AtChem/MCM modelling for all other VOCs are reported in Fig. S3 to S7,† showing the time-series of VOCs and ozone consumption, and excited and stabilised CIs production. In general, the results of the AtChem/MCM model show that the ozonolysis reaction is very fast under our experimental conditions and the excited CIs decompose quickly in the flow tube so that their concentrations (mostly below ppb levels) are estimated to be far below detection limits at the mixing point with DMPO (after 3 seconds reaction time) for all experiments. On the contrary, detectable amounts in the ppb range of SCIs are still present at the end of the flow tube and they can therefore be trapped by the DMPO. Our results show that the method used here is suitable for the detection of SCIs in laboratory experiments. Further studies are needed to investigate the possibility of detecting excited CIs.

A comparison between theoretically expected concentrations of SCIs and experimental measurements of CI–DMPO adducts is reported in Table 2. The results show that measured concentrations of CI–DMPO adducts are at least one order of magnitude lower than the modelled concentrations of SCIs from the AtChem/MCM model. This may be explained with wall losses in the systems, which were not estimated. The efficiency of the spin trapping reaction should be good, as ozone was generally the limiting reagent, to minimise reaction with DMPO, and DMPO was at least 4 orders of magnitude in excess with respect to the CIs. Nevertheless, reaction kinetics of SCIs with DMPO are unknown and this could also partly explain the discrepancy between experimental measurements and modelling results. The discrepancy is larger for the CI_C9βpin_ for which the reaction with DMPO has a larger energy barrier decreasing the adduct formation rate (Fig. 6). In addition, the fragmentation pattern of CIs–DMPO adducts in the PTR-ToF-MS is unknown which can lead to an underestimation of CIs–DMPO concentration. Nevertheless, MCM is not a fully explicit mechanism and, for example, does not include self-reaction of SCIs, overestimating SCI concentrations.^41^

The measured ratios of CIs produced from the different precursors do not match well the theoretically calculated distribution from the AtChem/MCM model. For example, for the ozonolysis of β-pinene, the MCM model predicts a distribution of 59% of SCI_C1_ and 41% of SCI_C9βpin_ while the experimentally measured distribution is 80% of CI_C1_–DMPO and 20% of CI_C9βpin_–DMPO. This large discrepancy can be explained by considering the stability of the CI–DMPO adducts. The results of the DFT calculations show that the CI_C1_–DMPO is more stable than the CI_C9βpin_–DMPO. In addition larger CIs, like CI_C9βpin_ and CI_C7Ar_, were generally measured at lower concentrations than expected from the modelling which might be because of the low volatility of these large CIs resulting potentially in wall losses. However, the temperature of the line after the DMPO mixing point was kept at 85 °C to minimise condensation on the walls. Volatility-related artefacts could help in explaining why there is a better match between measurements and modelling results for smaller CIs compared to the large β-pinene CI_C9βpin_ and styrene CI_C7Ar_.

In future studies, experimental strategies to improve quantification could aim to account for both the stability of the CI–DMPO adducts, as adducts with lower stability tends to be more underestimated, and their volatility because some of the adducts have rather high molecular weights, and partitioning into the condensed phase may be non-negligible. This seems to be suggested also by the memory effects in the system (*i.e.*, the slow decrease of signal after the production of ozone in the flow tube is turned off, Fig. 5 and 6).

In the case of limonene, the MCM reaction scheme considers only the addition of ozone to the double bond in the *endo* position as the *endo*-double bond is more reactive than the *exo*-double bond (95 : 5).^38^ However, in our experiments, all CIs from the reaction of ozone with both the *endo*- and *exo*-double bond have been detected, with ozone being in excess compared with limonene. However, surprisingly the CI–DMPO from the reaction of ozone with the *exo*-double bond were detected at higher concentrations than the CI–DMPO from reaction at the *endo*-double bond. This may be explained by different stabilities and volatilities of the CI–DMPO adducts.

Second generation CIs from the ozonolysis of the olefinic oxidation products from limonene were not detected in this series of experiments, which is consistent with theoretical calculations (Fig. S8†) that predict concentrations orders of magnitude below detection limits. However, second generation CIs may be produced in the condensed phase as the oxidation products from limonene ozonolysis are likely to partition efficiently into the particle phase.

**Fig. 8:**
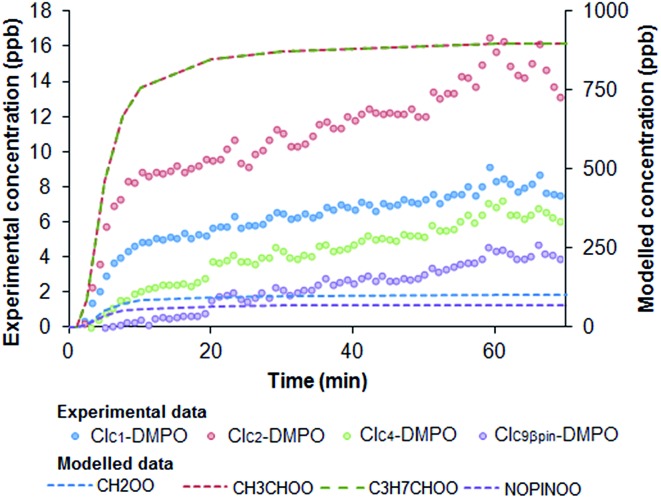
Time evolution of CIs–DMPO adducts (experimental) and SCIs (modelled) in the ozonolysis of a VOC mixture of β-pinene and *cis*-2-hexene. AtChem/MCM model simulation was run at different initial concentrations of ozone to simulate the warming-up time of the UV lamp used to generate ozone.

Simulations of the AtChem/MCM model in which ozone concentration was changed according to the output of the UV lamp (Fig. S2†) show that the initial increase of the concentrations of CIs–DMPO adducts before reaching a plateau is mainly due to the warming up time of the UV lamp before it reaches a constant ozone output (Fig. 8).

## 4. Conclusions

We identified and estimated concentrations of a series of SCIs from the ozonolysis of both biogenic and anthropogenic VOCs by using stabilisation with the spin trap DMPO and analysis with PTR-ToF-MS. This method proved to be applicable to SCIs with a wide range of structures and allowed us to measure SCIs that were otherwise out of reach for techniques used in previous studies. In addition, for the first time, it was possible to study an even more complex reaction system consisting of more than one olefinic precursor with the simultaneous detection of four SCIs.

The method has great potential to be used for the quantification of SCIs in laboratory experiments although specific calibration procedures need to be developed to improve accuracy, including assessment of instrumental response at high VOC concentration and estimating fragmentation patterns of CI–DMPO adducts and reaction kinetics between CIs and spin traps. The integrated approach used in this study combining DFT calculations to determine the stability of the CI–DMPO adducts, experimental measurements and MCM modelling reveals the importance of assessing the stability of adducts to aid the interpretation of measurement results but also volatility in the case of larger SCIs. In this context, synthesis of more volatile nitrone spin traps may help to overcome this weakness. The suitability of the technique to characterise excited CIs will need to be determined in future studies.

According to recent estimates, ambient SCI concentration in Hyytiälä (Finland) in the summer of 2010 was about 5 × 10^4^ molecules per cm^3^ with an order of magnitude uncertainty.^16^ Such concentration levels are about four to five orders of magnitude lower than the detection limits of our instrument^18^ and extremely challenging for any instrumental technique currently available even with an *ad hoc* pre-concentration method. Nevertheless, our new technique is uniquely capable of quantifying many different SCIs simultaneously and thus provides a significant step towards studying SCIs in realistic, complex reaction mixtures in the laboratory.

The method proposed here can be used for direct kinetic measurements, however, the reactivity of the spin trap toward ozone represents a limiting factor on the range of reaction conditions that can be tested. Generation of CIs in ozone-free conditions, *e.g. via* a diiodoalkane photolysis method,^6^ would allow us to perform kinetic experiments and compare our method with other measurement methods like PIMS and IR/UV spectroscopy.

Recently, extremely low volatile organic compounds (ELVOC) have been discovered, which irreversibly condense into the particle phase enhancing, and in some cases dominating, the early stage of atmospheric aerosol formation (nucleation), constituting a crucial link between new particle formation and cloud condensation nuclei formation.^42^,^43^ The suggested formation pathway of ELVOC from biogenic VOCs relies on initiation *via* ozonolysis of terpenes, and therefore CI formation, followed by an autoxidation process involving molecular oxygen (vinylhydroperoxide pathway).^42^,^44^ Measurement of terpene derived CIs using spin traps as CI scavengers may help in mechanistic studies to elucidate ELVOC formation mechanism, and their role in particle nucleation.

## Supplementary Material

Supplementary information
