# Supplementary material for: Detection and identification of Criegee intermediates from the ozonolysis of biogenic and anthropogenic VOCs: comparison between experimental measurements and theoretical calculations
Source: Faraday Discuss. 2017 Mar 6;200:559–78. doi: 10.1039/c7fd00025a (PMC5708353; doi:10.1039/c7fd00025a)
Supplement: Supplementary file 1 [file FD-200-C7FD00025A-s001.pdf]

Electronic Supplementary Material (ESI) for Faraday Discussions.
This journal is © The Royal Society of Chemistry 2017

Supplementary information for

Detection and identification of Criegee intermediates from the ozonolysis of biogenic and anthropogenic VOCs: comparison between experimental measurements and theoretical calculations

Chiara Giorio[1a]*, Steven J. Campbell[1], Maurizio Bruschi[2], Alexander T. Archibald[1], Markus Kalberer[1]*

[1] Department of Chemistry, University of Cambridge, Lensfield Road, Cambridge, CB2 1EW, United Kingdom
[2] Dipartimento di Scienze dell'Ambiente e del Territorio e di Scienze della Terra, Università degli Studi di Milano Bicocca, Piazza della Scienza 1, Milano, 20126, Italy
[a] now at: Aix-Marseille Université, CNRS, LCE UMR 7376, 13331 Marseille, France

*correspondence to: chiara.giorio@atm.ch.cam.ac.uk; markus.kalberer@atm.ch.cam.ac.uk

Contents

13 pages

13 figures

2 tables

S1. Experimental part

S1.1 Schematic of the flow tube and ozonolysis reaction time

Ozone has been introduced into the flow tube reactor via an 8 mm diameter glass tube of variable length (Figure S1) that allows to vary the reaction time. The outlet of the flow tube is then mixed into a T connection with DMPO in N_2 carrier gas. In the configuration used in this study, a residence time in the flow tube of 45 seconds represents the upper limit of the reaction time under the assumption that ozone is immediately mixed with the olefinic precursor at the point in which ozone is injected. However, in our experimental conditions the flow rates of the olefinic precursor (injected at the beginning of the large flow tube) and of ozone (injected through a much narrower tube close to the end of the flow tube) are equal and therefore ozone velocity is about 50 times higher than that of the VOCs. This means that the mixing is turbulent and a better approximation of the actual reaction time is represented by the lower limit that considers that the reaction is happening only at the centre of the flow tube (hatched rectangle in Figure S1).

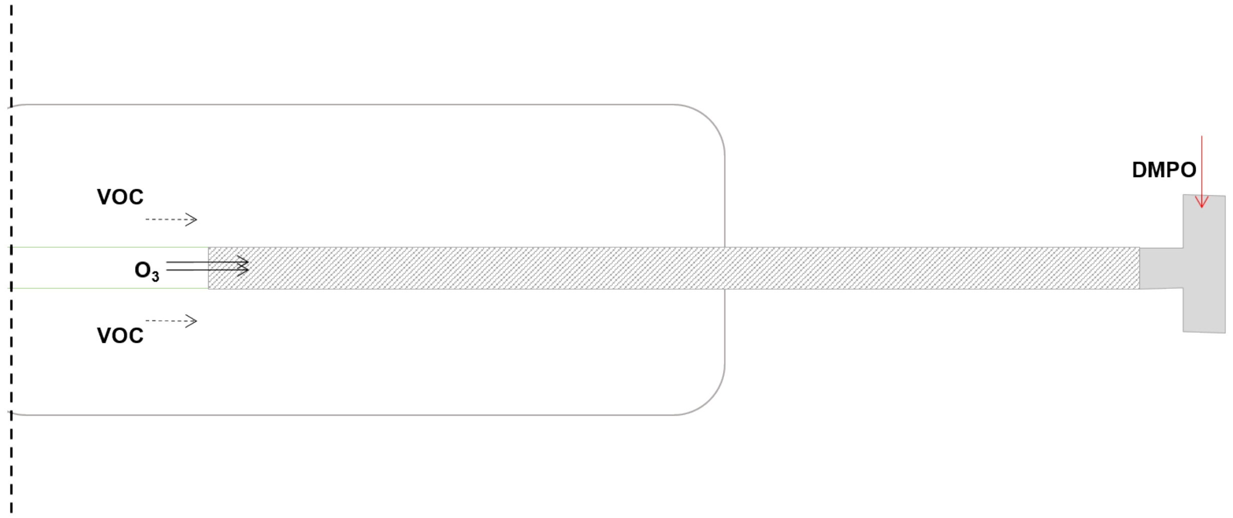

Figure S 1. Schematic of the final section of the flow tube showing the point in which O_3 is mixed with the olefinic VOC and the ozonolysis reaction occurs up to the mixing point with DMPO. The hatched rectangle shows the area considered for calculation of the residence time in the flow tube.

S1.2 Measurement of the ozone output from the UV lamp in our experimental set-up

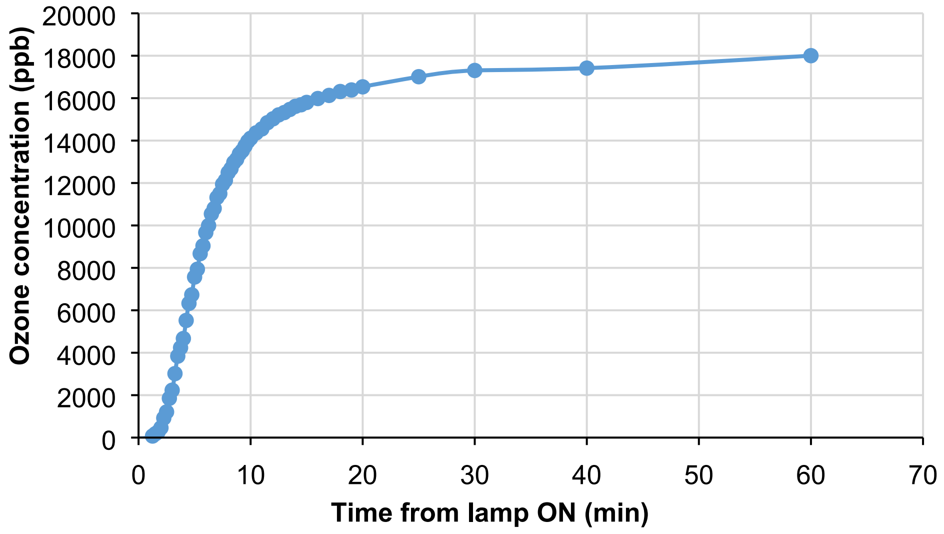

Figure S 2. Concentration of ozone produced by the UV lamp in our experimental conditions showing the warming up period of the lamp of about 20-30 minutes.

S2. Results of AtChem/MCM modelling

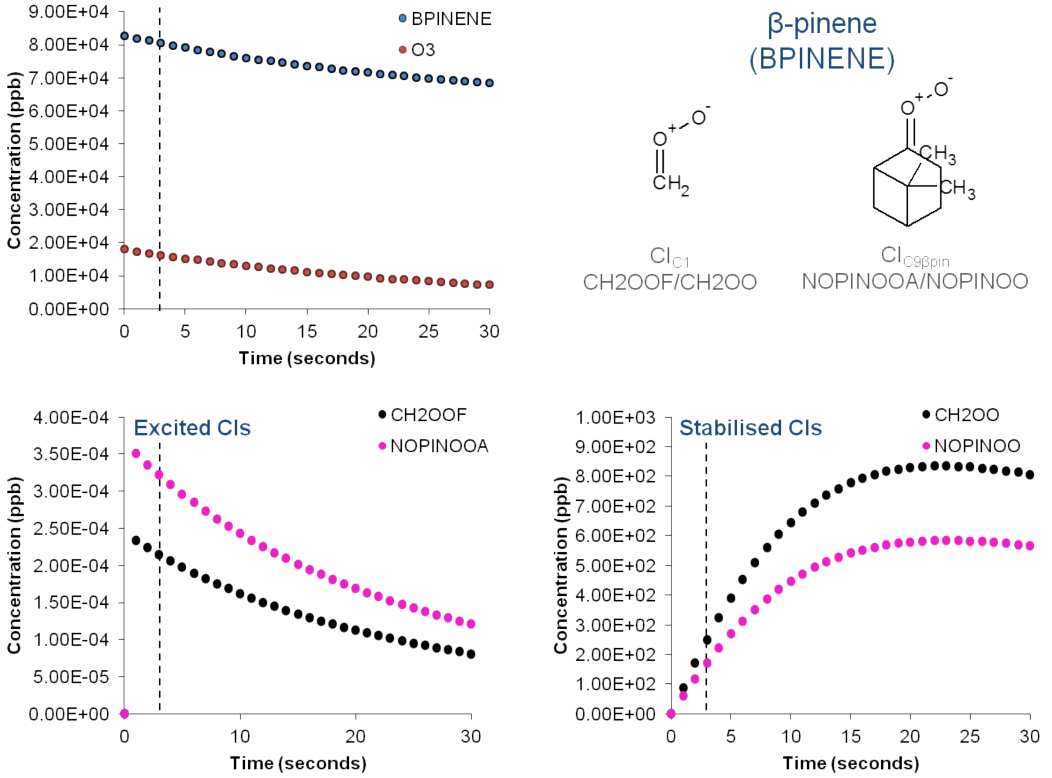

Figure S 3. Time evolution of precursors, excited CIs and stabilised CIs in the ozonolysis of β-pinene determined by the MCM model simulating our experimental conditions. Dashed vertical bars indicate reaction time in our steady-state flow tube experiments.

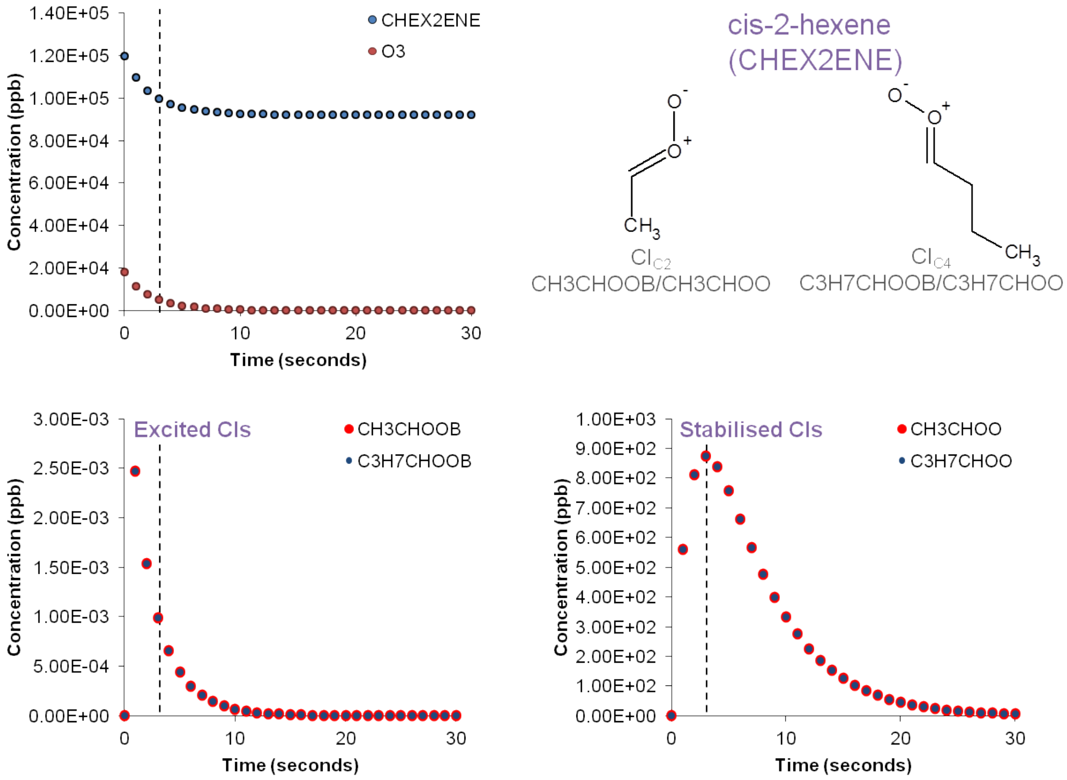

Figure S 4. Time evolution of precursors, excited CIs and stabilised CIs in the ozonolysis of cis-2-hexene determined by the MCM model simulating our experimental conditions. Dashed vertical bars indicate reaction time in our steady-state flow tube experiments.

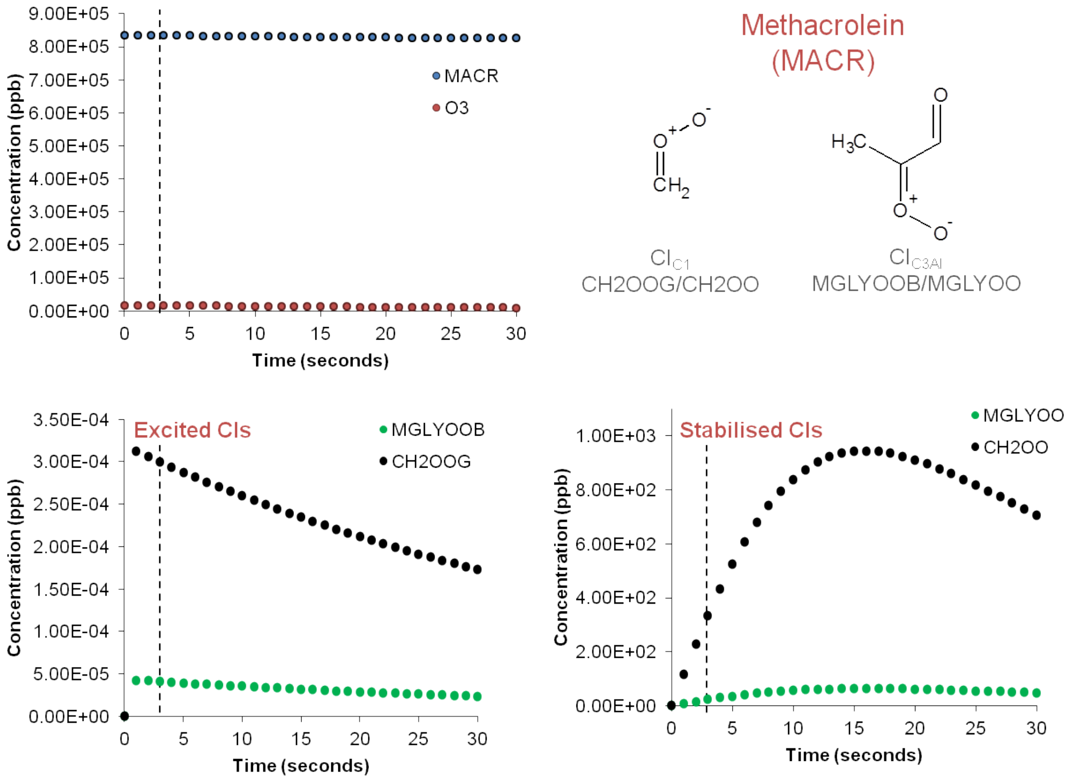

Figure S 5. Time evolution of precursors, excited CIs and stabilised CIs in the ozonolysis of methacrolein determined by the MCM model simulating our experimental conditions. Dashed vertical bars indicate reaction time in our steady-state flow tube experiments.

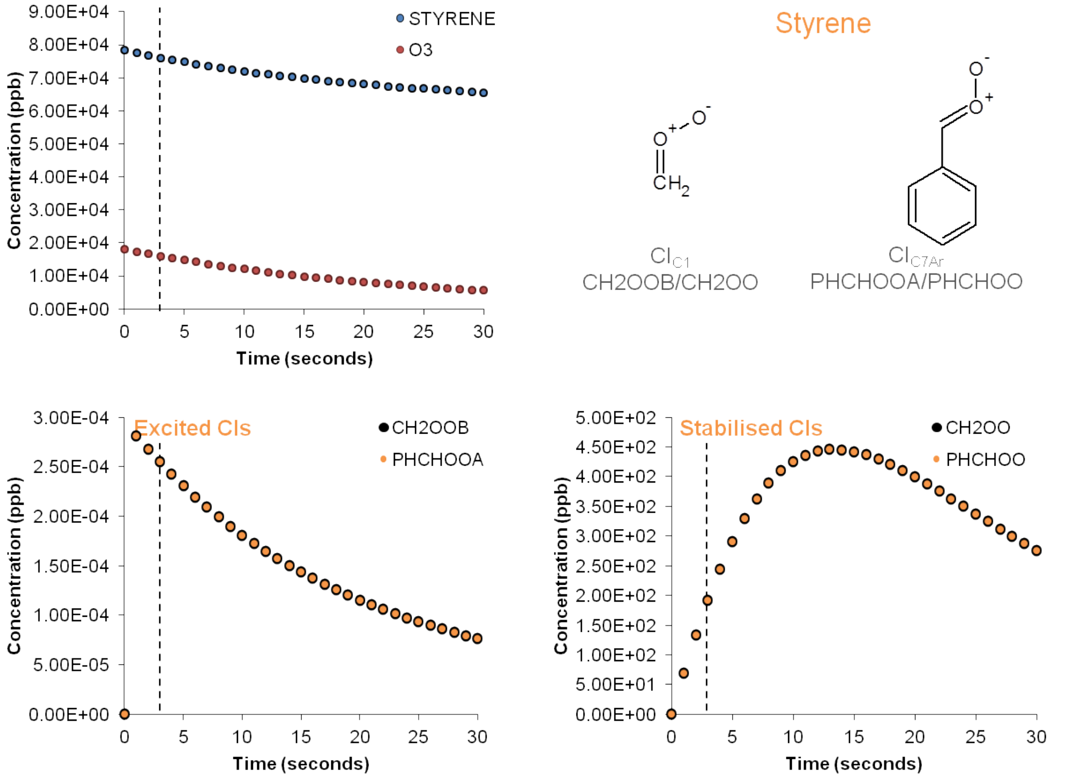

Figure S 6. Time evolution of precursors, excited CIs and stabilised CIs in the ozonolysis of styrene determined by the MCM model simulating our experimental conditions. Dashed vertical bars indicate reaction time in our steady-state flow tube experiments.

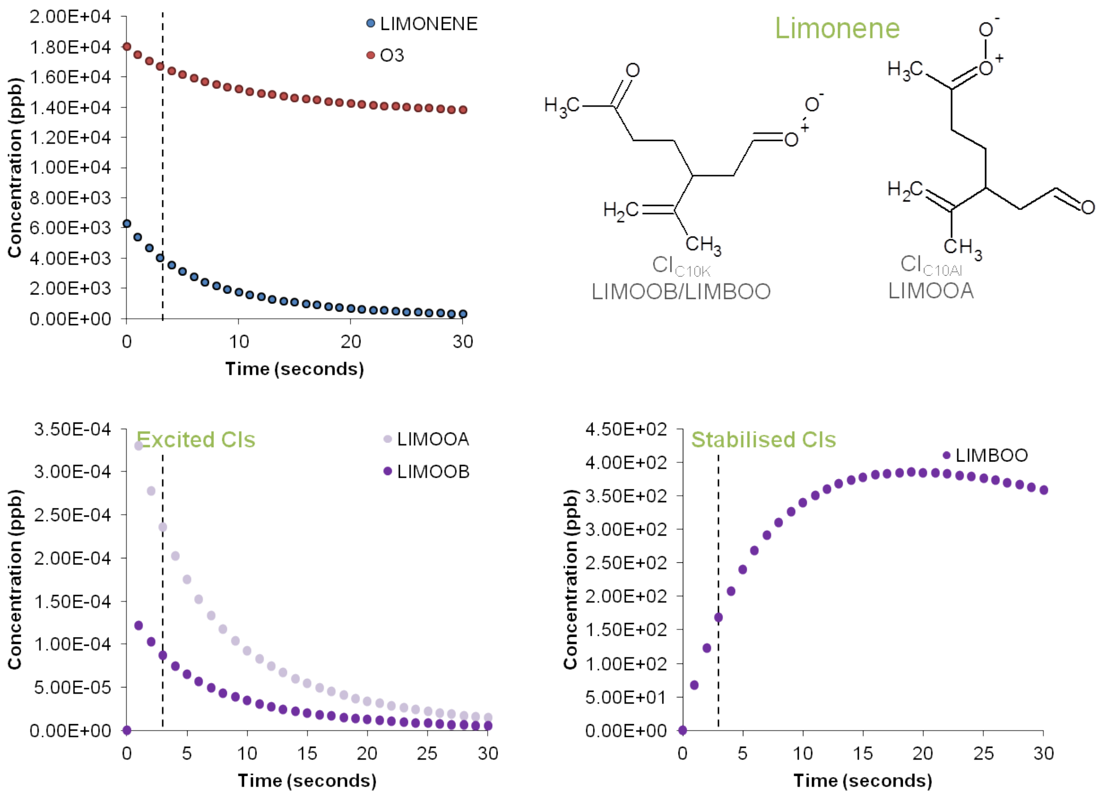

Figure S 7. Time evolution of precursors, excited CIs and stabilised CIs in the ozonolysis of limonene determined by the MCM model simulating our experimental conditions. Dashed vertical bars indicate reaction time in our steady-state flow tube experiments.

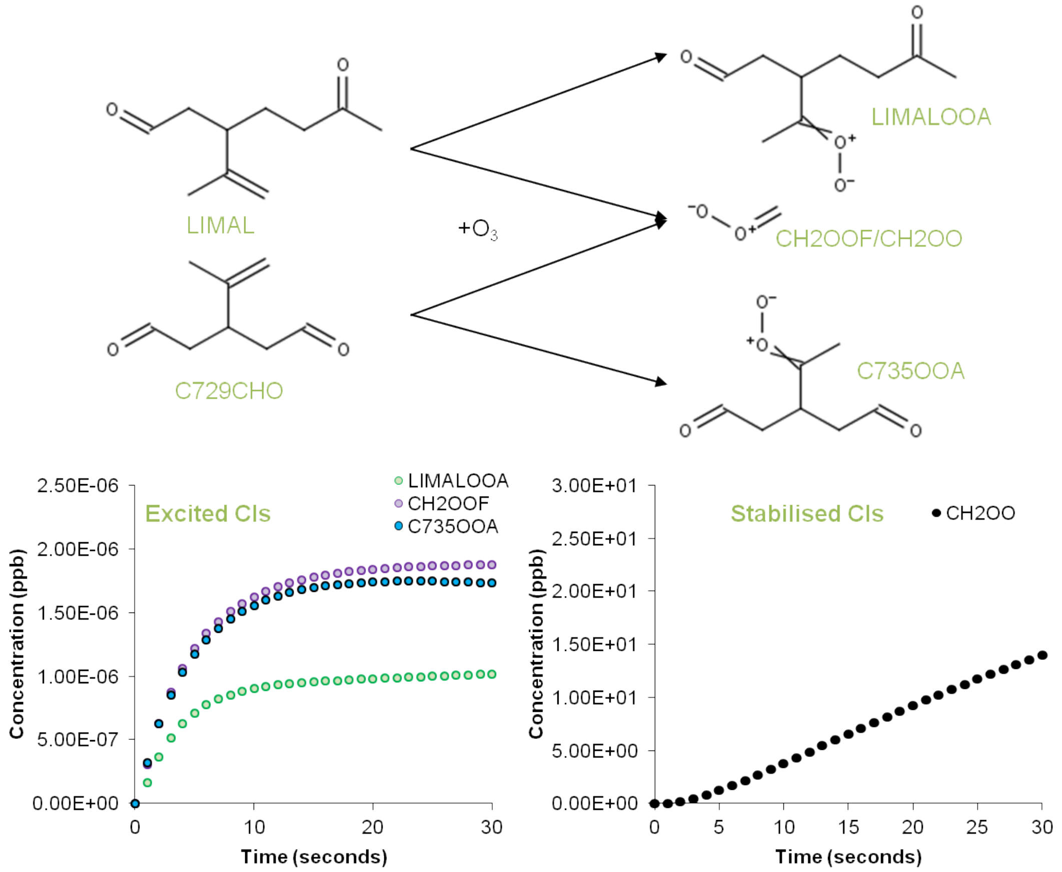

Figure S 8. Reaction scheme and time evolution of second generation excited CIs and stabilised CIs in the ozonolysis of limonene determined by the MCM model simulating our experimental conditions.

S3. DFT calculations

Table S 1. DFT energies (in Hartree) and thermochemical corrections to absolute enthalpies (H, in kcal/mol) for all of the species investigated in this work calculated using the BP86 and B3LYP functionals. The labels vdw-CI$_X$-DMPO, TS-CI$_X$ –DMPO and CI$_X$-DMPO (X= C1, C2, C4, C3Al, C9βpin) refer to van der Waals pre-reactive complexes, transition states, and the reaction products, respectively. The strings (syn) and (anti) in the labels indicate the initial conformation of the CIs, whereas the strings (RR) and (RS) indicate the absolute configuration of the two stereogenic centres generated in the products by the ring closure of CIs with DMPO. Finally, the string 5R attached to the labels refers to the 5-membered ring adducts.

	BP86		B3LYP	
	**Energy**	**H**	**Energy**	**H**
**CI$_{C1}$**	-189.68197749940	21.36	-189.56608692357	21.94
**DMPO + CI$_{C1}$**	-555.01039949024	126.93	-554.64398177637	130.24
**vdw-CI$_{C1}$-DMPO**	-555.01933673780	128.41	-554.65646613800	131.87
**TS-CI$_{C1}$-DMPO**	-555.01468412516	128.12	-554.65561118717	131.76
**CI$_{C1}$-DMPO**	-555.07308831951	130.12	-554.71506920591	133.82
**CI$_{C1}$-DMPO-5R**	-555.04703275616	129.61	-554.683936602	133.60
**CI$_{C2}$(syn)**	-229.02511305612	38.65	-228.880735450	39.66
**DMPO + CI$_{C2}$(syn)**	-594.35353504696	144.21	-593.958630303	147.96
**VDW-CI$_{C2}$(syn)-DMPO**	-594.36111404340	146.14	-593.969721533	149.97
**TS-CI$_{C2}$(syn)-DMPO**	-594.34814898187	145.82	-593.958868203	149.76
**CI$_{C2}$(SR)-DMPO**	-594.40278544186	147.69	-594.016522730	151.81
**CI$_{C2}$(anti)**	-229.02246054661	39.22	-228.878675730	40.20
**DMPO + CI$_{C2}$(anti)**	-594.35088253745	144.79	-593.956570583	148.50
**VDW-CI$_{C2}$(anti)-DMPO**	-594.35849683240	146.23	-593.968284404	150.01
**TS-CI$_{C2}$(anti)-DMPO**	-594.35244515432	145.81	-593.964380148	149.77
**CI$_{C2}$(RR)-DMPO**	-594.40817229023	147.72	-594.022152751	151.83
**CI$_{C2}$(RR)-DMPO-5R**	-594.38261459064	146.81	-593.988990663	151.40
**CI$_{C4}$(syn)**	-307.67906715252	75.68	-307.478302874	77.59
**DMPO + CI$_{C4}$(syn)**	-673.00748914336	181.24	-672.556197727	185.89
**vdw-CI$_{C4}$(syn)-DMPO**	-673.01205253420	182.01	-672.565426987	186.72
**TS-CI$_{C4}$(syn)-DMPO**	-673.00099221696	181.54	-672.555009986	187.01
**CI$_{C4}$(SR)-DMPO**	-673.05474023645	183.82	-672.611734008	188.83
**CI$_{C4}$(anti)**	-307.67528323976	75.54	-307.474896944	77.42
**DMPO + CI$_{C4}$(anti)**	-673.00370523060	181.10	-672.552791797	185.72

**vdw-CI$_{C4}$(anti)-DMPO**	-673.00940307680	182.40	-672.562848272	187.08
**TS-CI$_{C4}$(anti)-DMPO**	-673.00333738532	181.93	-672.558740053	186.79
**CI$_{C4}$(RR)-DMPO**	-673.05966652739	183.90	-672.617029816	188.90
**CI$_{C4}$(RR)-DMPO-5R**	-673.03285895590	182.97	-672.582643115	188.35
				\
**CI$_{C3Al}$(syn)**	-342.40508943203	46.07	-342.19458759906	47.27
**DMPO + CI$_{C3Al}$(syn)**	-707.73351142287	151.64	-707.27248245186	155.57
**vdw-CI$_{C3Al}$(syn)-DMPO**	-707.73828050660	152.99	-707.28049788530	156.99
**TS-CI$_{C3Al}$(syn)-DMPO**	-707.71498330624	152.52	-707.26195558854	156.42
**CI$_{C3Al}$(RR)-DMPO**	-707.76437787700	153.62	-707.31560624317	158.07
**CI$_{C3Al}$(anti)**	-342.39404549779	45.97	-342.18512800976	47.17
**DMPO + CI$_{C3Al}$(anti)**	-707.72246748863	151.54	-707.26302286256	155.48
**vdw-CI$_{C3Al}$(anti)-DMPO**	-707.73457311570	152.89	-707.27777834710	156.90
**TS-CI$_{C3Al}$(anti)-DMPO**	-707.72207888862	152.38	-707.26926796583	156.96
**CI$_{C3Al}$(SR)-DMPO**	-707.77068640555	153.90	-707.32087364105	158.21
**CI$_{C3Al}$(RR)-DMPO-5R**	-707.74239011534	153.50	-707.28440450926	158.13
**CI$_{C9βpin}$(anti)**	-501.87605827546	137.34	-501.53402337817	140.92
**DMPO + CI$_{C9βpin}$(anti)**	-867.20448026630	242.91	-866.61191823097	249.23
**vdw-CI$_{C9βpin}$(anti)-DMPO**	-867.20966062030	244.25	-866.59489872625	250.29
**TS-CI$_{C9βpin}$(anti)-DMPO**	-867.18321142654	243.80	-866.59478879625	250.29
**CI$_{C9βpin}$(RR)-DMPO**	-867.23883564418	245.34	-866.65341721554	252.02
**CI$_{C9βpin}$(syn)**	-501.87230381852	137.48	-501.53027166177	140.49
**DMPO + CI$_{C9βpin}$(syn)**	-867.20072580936	243.05	-866.60816651457	248.79
**vdw-CI$_{C9βpin}$(syn)-DMPO**	-867.20702411540	244.49	-866.61754549743	250.53
**TS-CI$_{C9βpin}$(syn)-DMPO**	-867.18589340982	243.77	-866.59619578036	250.28
**CI$_{C9βpin}$(SR)-DMPO**	-867.23983887076	245.34	-866.65410809670	252.03

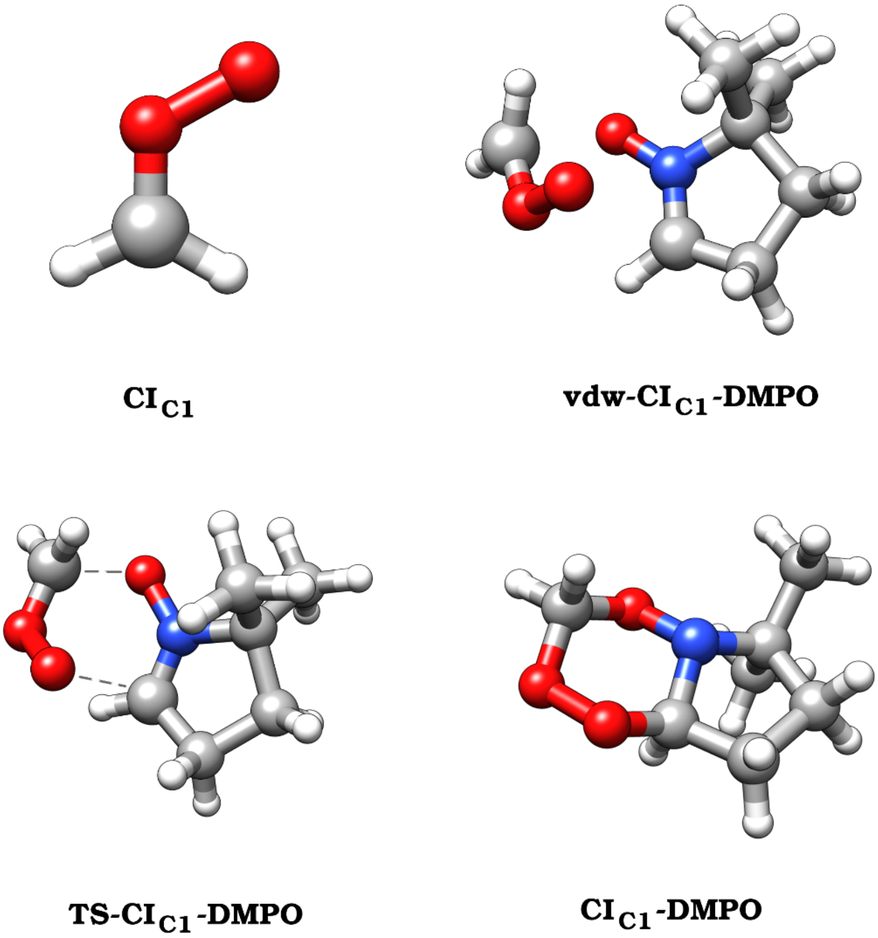

Figure S 9. DFT-B3LYP optimized structures of CI_{C1}, and the relevant species formed along the pathway of the reaction of CI_{C1} and DMPO. For the labels, see the caption of Table S1

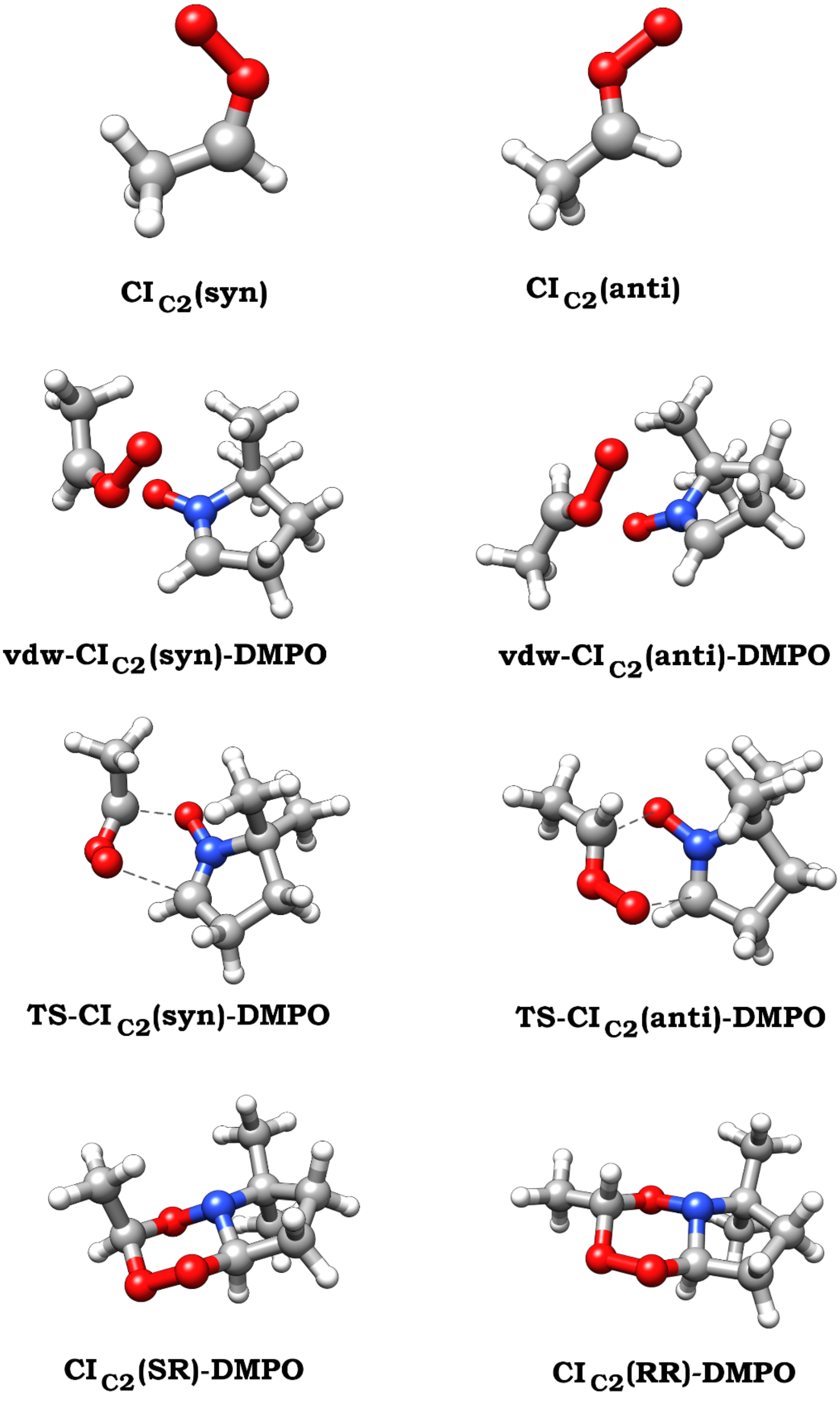

Figure S 10. DFT-B3LYP optimized structures of CI_{C2}, and the relevant species formed along the pathway of the reaction of CI_{C2} and DMPO. For the labels, see the caption of Table S1

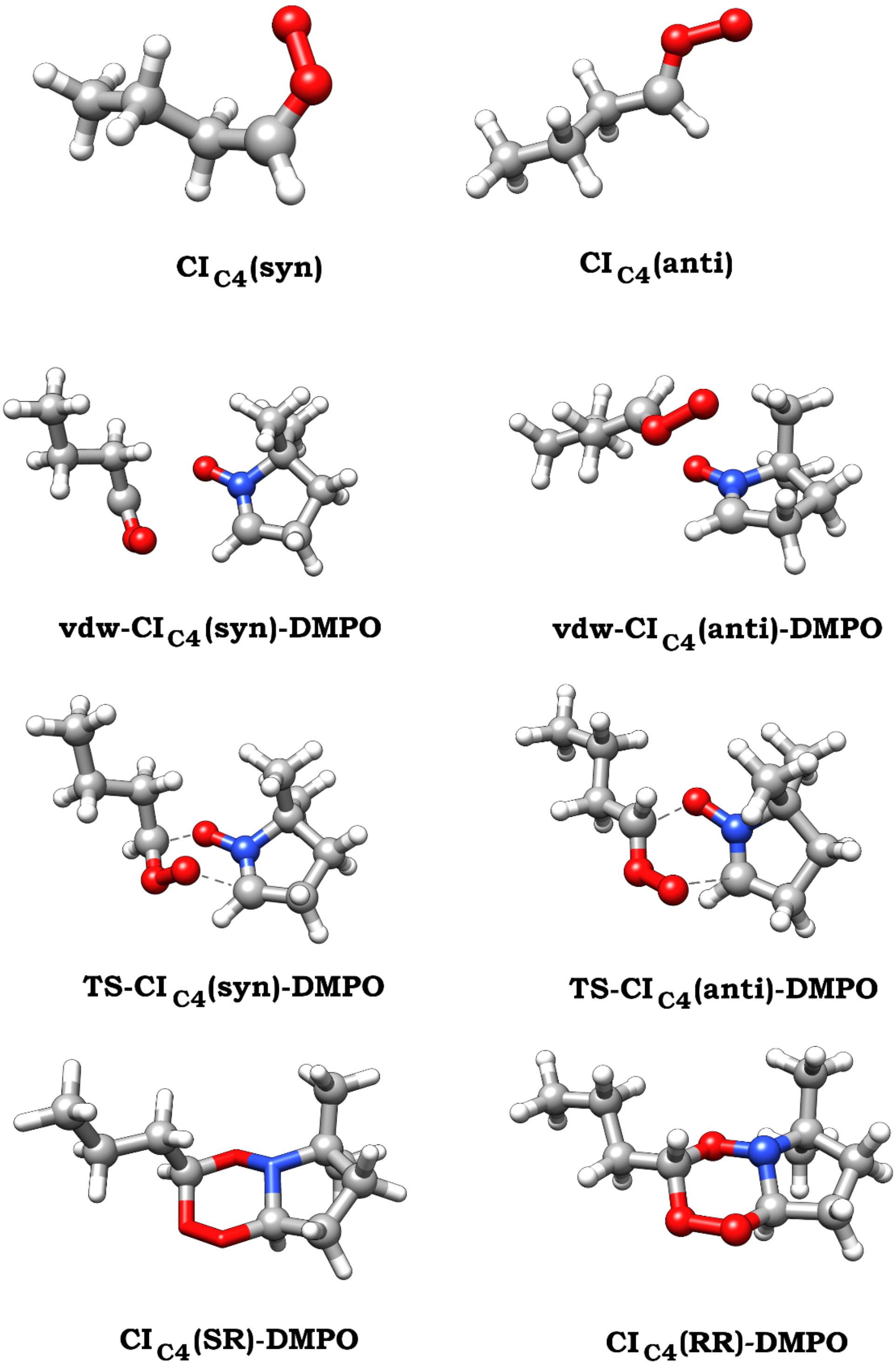

Figure S 11. DFT-B3LYP optimized structures of CI_{C4}, and the relevant species formed along the pathway of the reaction of CI_{C4} and DMPO. For the labels, see the caption of Table S1

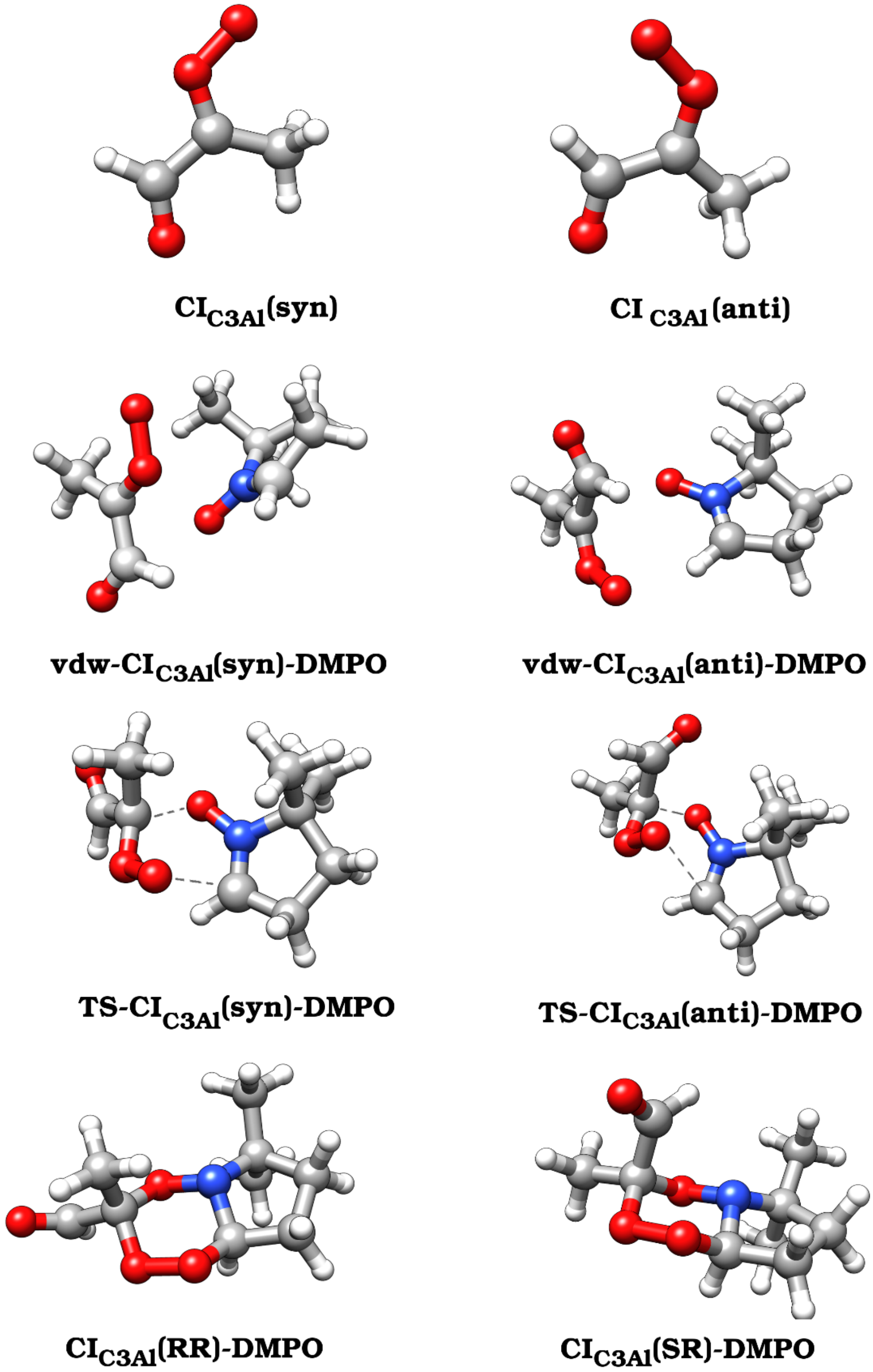

Figure S 12. DFT-B3LYP optimized structures of CI$_{C3Al}$, and the relevant species formed along the pathway of the reaction of CI$_{C4}$ and DMPO. For the labels, see the caption of Table S1

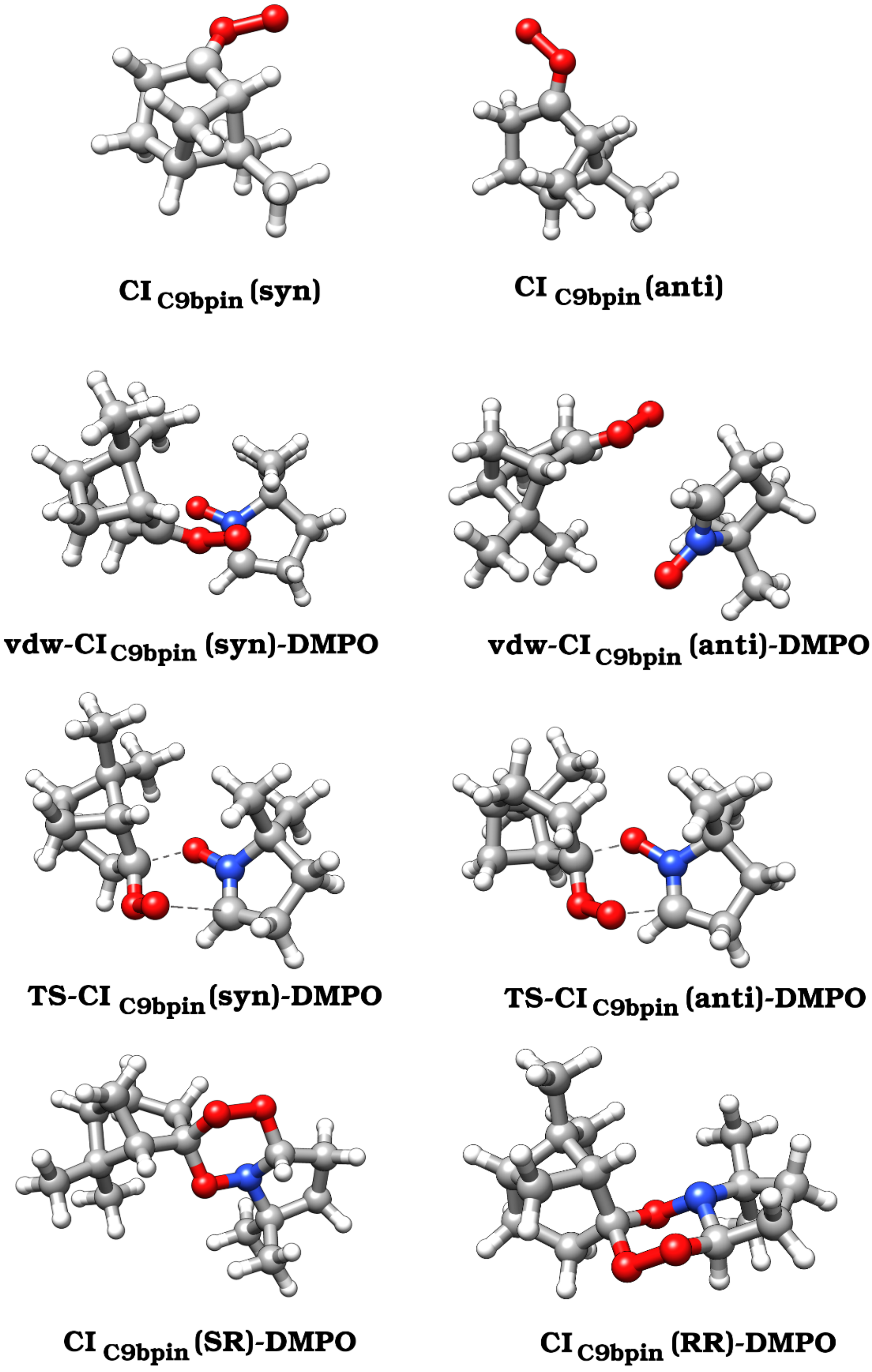

Figure S 13. DFT-B3LYP optimized structures of $CI_{9\beta pin}$, and the relevant species formed along the pathway of the reaction of $CI_{C9\beta pin}$ and DMPO. For the labels, see the caption of Table S1

S4. Theoretical calculations of proton transfer reaction rate coefficients

Proton transfer reaction rates for DMPO and all CIs-DMPO adducts have been calculated using the Langevin model, describing a long range interaction between a point charge and a polarisable molecule,[1] the average-dipole-orientation theory (ADO) which considers also the permanent dipole moment of the neutral organic species,[1] and the refined ADO theory parametrised by Su and Chesnavich[2] to account for the kinetic energy dependence of the rate constant across a wide range of temperatures. Average static polarizability and the dipole moment of the DMPO and CIs-DMPO adducts were calculated using the B3LYP/def-TZVP level of theory as described in the main text (section 2.4). Details on the calculations of proton transfer rate coefficients using the three different theories can be found elsewhere.[3,4] Rate constants of DMPO and CIs-DMPO adducts for T=300K and a drift tube temperature of 363.15K, as used in this study, are reported in Table S2. An average of k_{cap} at 363.15K for the RR and SR orientations of each molecule and an average of the k_{cap} for CI_{C10Al} and CI_{C10K} were used for quantification of all CIs-DMPO adducts.

Table S2. Polarizability, dipole moment, Langevin collision rate constant (k_L), ADO collision rate constant (k_{ADO}) and ion-polar molecule capture collisions rate constant (k_{cap}) at 300K and 363.15K (operating drift tube temperature in our experiments).

Compound	α (Å^3)	μ_D (D)	T (K)	k_L (x10^{-9} cm^3 s^{-1})	k_{ADO} (x10^{-9} cm^3 s^{-1})	k_{cap} (x10^{-9} cm^3 s^{-1})
**DMPO**	12.06	4.07	300	2.02	4.34	2.77
			363.15	2.02	4.13	2.75
**CI_{C3Al} (RR)-DMPO**	18.42	4.8	300	2.41	5.04	3.37
			363.15	2.41	4.80	3.35
**CI_{C3Al} (SR)-DMPO**	18.49	4.84	300	2.42	5.07	3.37
			363.15	2.42	4.83	3.35
**C_{9bpin} (RR)-DMPO**	27.55	2.144	300	2.92	3.79	3.25
			363.15	2.92	3.71	3.22
**C_{9bpin} (SR)-DMPO**	27.38	2.073	300	2.91	3.74	3.21
			363.15	2.91	3.67	3.19
**CI_{C1} (R)-DMPO**	14.66	2.558	300	2.18	3.44	3.01
			363.15	2.18	3.33	2.95
**CI_{C4} (RR)-DMPO**	20.27	2.378	300	2.53	3.61	3.07
			363.15	2.53	3.51	3.03
**CI_{C4} (SR)-DMPO**	19.96	2.283	300	2.51	3.53	3.01
			363.15	2.51	3.44	2.97
**CI_{C2} (RR)-DMPO**	16.61	2.294	300	2.31	3.37	2.89
			363.15	2.31	3.28	2.85
**CI_{C2} (SR)-DMPO**	16.35	2.400	300	2.29	3.43	2.94
			363.15	2.29	3.33	2.90
**CI_{C7Ar} (RR)-DMPO**	24.67	2.339	300	2.77	3.78	3.22
			363.15	2.77	3.69	3.19
**CI_{C9lim} (RR)-DMPO**	28.40	2.068	300	2.96	3.78	3.26
			363.15	2.96	3.71	3.24
**CI_{C10Al} (RR)-DMPO**	34.45	4.921	300	3.28	5.82	4.78
			363.15	3.28	5.59	4.74
**CI_{C10K} (RR)-DMPO**	30.95	4.847	300	3.08	5.59	4.48
			363.15	3.08	5.36	4.45

References

1 R. S. Blake, P. S. Monks and A. M. Ellis, *Chem. Rev.*, 2009, **109**, 861–896.

2 T. Su and W. J. Chesnavich, *J. Chem. Phys.*, 1982, **76**, 5183–5185.

3 L. Cappellin, M. Probst, J. Limtrakul, F. Biasioli, E. Schuhfried, C. Soukoulis, T. D. Märk and F. Gasperi, *Int. J. Mass Spectrom.*, 2010, **295**, 43–48.

4 T. Su, *J. Chem. Phys.*, 1994, **100**, 4703–4703.
